# Characterization of the membrane proteome and N-glycoproteome in BV-2 mouse microglia by liquid chromatography-tandem mass spectrometry

**DOI:** 10.1186/1471-2164-15-95

**Published:** 2014-02-04

**Authors:** Dohyun Han, Sungyoon Moon, Yikwon Kim, Hophil Min, Youngsoo Kim

**Affiliations:** 1 Department of Biomedical Sciences, Seoul National University College of Medicine, 28 Yongon-Dong, Seoul 110-799, Korea; 2 Institute of Medical & Biological Engineering, Medical Research Center, Seoul National University College of Medicine, 28 Yongon-Dong, Seoul 110-799, Korea

**Keywords:** Microglia, Membrane proteome, N-glycoproteome, Proteomics, Crude membrane fractionation, FASP, N-glyco-FASP

## Abstract

**Background:**

Microglial cells are resident macrophages of the central nervous system and important cellular mediators of the immune response and neuroinflammatory processes. In particular, microglial activation and communication between microglia, astrocytes, and neurons are hallmarks of the pathogenesis of several neurodegenerative diseases. Membrane proteins and their N-linked glycosylation mediate this microglial activation and regulate many biological process including signal transduction, cell-cell communication, and the immune response. Although membrane proteins and N-glycosylation represent a valuable source of drug target and biomarker discovery, the knowledge of their expressed proteome in microglia is very limited.

**Results:**

To generate a large-scale repository, we constructed a membrane proteome and N-glycoproteome from BV-2 mouse microglia using a novel integrated approach, comprising of crude membrane fractionation, multienzyme-digestion FASP, N-glyco-FASP, and various mass spectrometry. We identified 6928 proteins including 2850 membrane proteins and 1450 distinct N-glycosylation sites on 760 N-glycoproteins, of which 556 were considered novel N-glycosylation sites. Especially, a total of 114 CD antigens are identified via MS-based analysis in normal conditions of microglia for the first time. Our bioinformatics analysis provides a rich proteomic resource for examining microglial function in, for example, cell-to-cell communication and immune responses.

**Conclusions:**

Herein, we introduce a novel integrated proteomic approach for improved identification of membrane protein and N-glycosylation sites. To our knowledge, this workflow helped us to obtain the first and the largest membrane proteomic and N-glycoproteomic datesets for mouse microglia. Collectively, our proteomics and bioinformatics analysis significantly expands the knowledge of the membrane proteome and N-glycoproteome expressed in microglia within the brain and constitutes a foundation for ongoing proteomic studies and drug development for various neurological diseases.

## Background

Microglia, which are representative immune cells in the relatively immune-privileged central nervous system (CNS), mediate immune and inflammatory responses in the brain [1,2]. In response to pathological events, such as immunological stimuli, neuronal injury, and tissue damage, microglia alter their morphology, migrate to lesion sites, and proliferate [2,3]. Proliferating microglia phagocytose pathogens, dying neuronal cells, lymphocytes, and other debris [3] and release a wide range of soluble factors, including cytokines, chemokines, and oxygen radicals, to maintain homeostasis in the microenvironment and to support injured neurons in the brain [3-5].

Due to their function in immune and inflammation responses in the brain, microglia are recently considered central mediators in various neurological diseases, such as HIV-1-associated dementia, Alzheimer disease (AD), Parkinson disease (PD), tumors, brain and spinal cord trauma, stroke, and autoimmune CNS disease [6,7].

Microglia communicate actively with neurons and astrocytes in the brain. This communication is essential for the maintenance of homeostasis in the brain and for appropriate immune responses to microenvironmental alterations [8]. Membrane proteins and their N-glycosylation mediate this communications, which regulates many functions, such as signal transduction, subcellular compartmentalization, membrane trafficking, and immune responses [9]. The molecular and cellular interactions between these proteins and their modification enable the cells to sense micro environmental variations and activate various mechanisms, including signaling pathways and transcriptional regulation of specific genes.

Based on the function in molecular and cellular interactions, membrane proteins and their glycosylation are considered significant with regard to disease markers and drug treatment targets, accounting for nearly 70% of pharmaceutical drug targets and biomarkers [10,11]. Thus, to understand microglial function in the microenvironment of the brain under normal and pathogenic conditions and develop therapeutic targets and biomarkers for neurological diseases, we must identify all such membrane proteins and N-glycoproteins. Although several proteomics studies have been performed in microglia [12-15], membrane proteins and N-linked glycoproteins have not been examined in microglia in great detail.

Mass spectrometry (MS)-based proteomic methods have emerged as powerful and universal tools to examine proteins and their properties [16]. Specifically, large-scale studies of membrane proteins and posttranslational modifications (PTMs) are core subjects in MS-based proteomics [17-19]. However, such studies continue to face technically challenges in determining the abundance, state of modification, and localization of proteins, due to several factors, including the solubility, abundance, digestion, and enrichment of membrane proteins and N-glycosylated peptides [20,21]. Thus, analytical strategies that are coupled with efficient methods, including enrichment, solubilization, and digestion of membrane proteins and N-glycoproteins, must be formulated.

Recently, sample preparation methods, such as filter-aided sample preparation (FASP) [22,23] and multienzyme digestion-FASP (MED-FASP) [24], have been developed to deplete sodium dodecyl sulfate (SDS), maximizing the solubility of membrane proteins. In addition, FASP-based N-glycopeptide enrichment (N-glyco-FASP) was developed to facilitate the identification of many glycoproteins [18]. These FASP-based methods have been used by several studies to improve the identification of total proteins, membrane proteins, N-glycoproteins, and other modifications [18,24-27].

In this study, we generated large-scale data on the membrane proteome and N-glycoproteome of the BV-2 microglia line by liquid chromatography-coupled tandem mass spectrometry (LC-MS/MS) without extensive peptide fractionation and examined the properties of the resulting proteins with regard to membrane localization and N-glycosylation. To derive a comprehensive membrane proteome and N-glycoproteome from BV-2 cells, we analyzed several replicates on various mass spectrometric instruments using multiple strategies, based on recent advances in proteomics technologies, such as crude membrane fractionation, FASP-based differential sample preparation, and N-glyco-FASP-based glycopeptide enrichment.

We present the most detailed microglia membrane proteome and N-glycoproteome dataset, resulting in the identification of 6928 unique protein groups and 1450 unique N-glycosites from 82 LC-MS runs. In addition, we characterized the membrane proteome and N-glycoproteome of BV-2 cells using various bioinformatics tools to classify functional groups and activities in microglia. This extensive profile, based on our novel approach, constitutes a reference repository of microglial membrane proteins and N-glycosylated proteins, which will be particularly useful for future functional and targeted proteomics studies in microglia.

## Methods

### Reagents and materials

HPLC-grade acetonitrile (ACN), HPLC-grade water, HPLC-grade methanol, hydrochloric acid (HCl), and sodium chloride (NaCl) were obtained from DUKSAN (Gyungkido, Korea). The BCA protein assay kit was purchased from Pierce (Hercules, CA), and Complete Protease Inhibitor Cocktail Mini Tablets were purchased from Roche (Mannheim, Germany). Dithiothreitol (DTT) and urea were purchased from AMRESCO (Solon, OH). PMSF, Sodium dodecyl sulfate (SDS) and Tris were purchased from USB (Cleveland, OH). Sequencing-grade modified trypsin and LysC were purchased from Promega Corporation (Madison, WI) and Wako (Osaka, Japan), respectively.

All other reagents—concanavalin A (ConA), wheat germ agglutinin (WGA), Ricinus communis agglutinin 120 (RCA120), 2-mercaptoethanol, ammonium bicarbonate (NH_4_HCO_3_), sucrose, EDTA, formic acid, iodoacetamide (IAA), trifluoroacetic acid (TFA), PNGase F, and stable isotope-labeled water (H_2_ ^18^O, 99% atom% ^18^O)—were purchased from Sigma-Aldrich (St. Louis, MO).

### Cell culture

Mouse microglia (BV-2 cell line) were maintained in DMEM complete media, containing 5% (v/v) heat-inactivated FBS, 4 mM glutamine, 100 U/mL penicillin, and 100 mg/mL streptomycin, at 37°C in a humidified atmosphere and 5% CO_2_.

### Crude membrane preparation

Crude membrane fractions were prepared using 4 different methods (CM method 1, CM method 2, KIT 1, and KIT 2). In CM method 1, membrane proteins were extracted as described with some modifications [28]. BV-2 cell pellets (1×10^7^ cells) were homogenized in 1 ml high-salt buffer (2 M NaCl, 10 mM HEPES-NaOH, pH 7.4, 1 mM EDTA, and 1X protease inhibitor cocktail) using a syringe with a 26_1/2_-gauge needle. The lysate was centrifuged at 17,500 *g* at 4°C for 30 min. The pellet was dissolved in 1 ml carbonate buffer (0.1 M Na_2_CO_3_, pH 11.3, 1 mM EDTA, and 1X protease inhibitor cocktail), incubated on ice for 30 min, and centrifuged at 17,500 g for 30 min at 4°C. Incubation and centrifugation were repeated with carbonate buffer. After centrifugation (17,500 g, 30 min at 4°C), the pellet was stored at -80°C until further analysis.

In CM method 2, membrane proteins were prepared as described with the following adaptations [29]. BV-2 cell pellets (1×10^7^ cells) were homogenized in 1 ml STM solution (0.25 M sucrose, 10 mM Tris–HCl, 1 mM MgCl_2_, and 1X protease inhibitor cocktail) using a syringe with a 26_1/2_-gauge needle. Nuclei and tissue debris were removed by centrifugation at 260 *g* for 5 min at 4°C. The supernatant was first centrifuged at 1500 *g* for 10 min at 4°C to pellet the crude membrane proteins. The pellet was then mixed with 0.7 ml STM solution and centrifuged at 16,000 *g* for 1 h at 4°C to purify the membrane pellet. The pellet was washed in 1 ml of 0.1 M Na_2_CO_3_, pH 11 overnight at 4°C. After centrifugation at 16,000 *g* for 1 h at 4°C, the purified membrane pellet was stored at -80°C for further processing. In contrast to other protocols [28,29], all crude membrane protein pellets were solubilized with strong SDS extraction buffer and subjected directly to digestion by FASP.

Crude membrane fractionation using Commercial kits (KIT 1 and KIT 2) were performed according to the manufacturer’s instructions.

### Protein multienzyme digestion by FASP (MED-FASP)

Multiple-step enzyme digestion using filter-aided sample preparation (MED-FASP) was performed as described [22-24,26]. First, pellets that contained the crude membrane fractions were resuspended in 200 μL strong SDS extraction buffer (100 mM Tris pH 7.4, 4% SDS, and 0.1 M DTT). Protein concentration was measured using a BCA assay kit. Approximately 200 μg of proteins was mixed with 200 μl UA solution (8 M urea in 0.1 M Tris/HCl pH 8.5), loaded onto a 30 k Microcon filtration unit (Millipore), and centrifuged at 14,000 *g* for 20 min at 20°C. The concentrates were diluted in the devices with 200 μL UA solution and centrifuged again.

Next, the concentrates were mixed with 200 μL IAA solution (50 mM iodoacetamide in UA solution), and incubated in the dark at room temperature (RT) for 30 min, and centrifuged for 15 min. Then, the concentrate was diluted with 200 μL UB solution (8 M urea in 0.1 M Tris/HCl, pH 8.5) and concentrated again. The concentrate with UB solution was washed 3 more times. After the flowthrough was discarded, 0.2 mL 50 mM ABC was added to the filter and centrifuged at 14,000 g for 15 min; this step was repeated 3 times.

Proteins were digested at 37°C overnight using LysC (enzyme-to-substrate ratio [w/w] of 1:50) or trypsin (enzyme to substrate ratio [w/w] of 1:100). After an overnight incubation at 37°C, the filtration unit was transferred to new collection tubes, and the digested peptides were collected by centrifugation for 20 min. Before the next digestion step, the filtration units were washed once with 40 μl UA solution and then with 2 40-μl washes with water. In the second digestion, 100 μl 50 mM ABC with trypsin (enzyme:protein ratio 1:100) was added to the filter units. After an overnight incubation at 37°C, the filtration unit was transferred to new collection tubes, and peptides were collected by centrifugation for 20 min. Finally, the peptides that were retained by the MWCO membrane in the filtration units were eluted with 50 μl 0.5 M NaCl to enhance the yield of the digested protein. All resultant peptides were acidified with 1% TFA and dried in a vacuum centrifuge.

Prior to LC-MS/MS analysis, all dried peptide mixtures were dissolved in 0.1% TFA and desalted using homemade StageTips, as follows. Self-packed C_18_ microcolumns were prepared by reversed-phase packing POROS 20 R2 material (Applied Biosystems, Foster City, CA) into 200-μl yellow pipette tips on top of C_18_ Empore disk membranes. The microcolumns were washed 3 times with 100 μl 100% ACN and equilibrated 3 times with 100 μl 0.1% TFA by applying air pressure from a syringe. After the samples were loaded, the microcolumns were washed 3 times with 100 μl 0.1% TFA, and peptides were eluted with 100 μl of a series of elution buffers, containing 0.1% TFA and 40%, 60%, and 80% ACN. All eluates were combined, dried in a vacuum centrifuge, and stored at -80°C until further analysis.

### Whole-cell lysate capture by N-glyco-FASP

N-glycosylated peptides were enriched by N-glyco-FASP [18]. In brief, BV-2 cells were cultured as described above, washed 3 times with PBS, harvested, and pelleted at 1000 *g* at 4°C. The pellets, containing 1×10^7^ cells, were dissolved in strong SDS extraction buffer. After measuring the total protein concentration by BCA assay, 300 μg of proteins was digested per the FASP protocol above. Digested peptides were mixed with lectin binding solution (20 mM Tris pH 7.6, 1 mM MnCl_2_, 1 mM CaCl_2_, and 0.5 M NaCl) and transferred to new TM-30 filter units (Microcon, Millipore).

Lectin solution, containing ConA (100 μg), WGA (100 μg), and RCA120 (80 μg), was added to the filter units. After one-hour incubation at room temperature, the unbound peptides were eluted by centrifugation at 14,000 *g* for 10 min. The captured fractions were washed several times with lectin binding solution and concentrated by centrifugation. To remove ordinary water (H_2_ ^16^O) and adjust the pH, concentrated peptides were washed twice with 50 μl ABC^18^O solution (40 mM ABC in H_2_ ^18^O). After the filter units were transferred to new collection tubes, PNGase F (2U in 40 μl ABC^18^O) was added to glycan-containing peptides in the filter units. The mixture was incubated at 37°C for 3 hr, and the deglycosylated peptides were eluted.

### Crude membrane fraction capture by N-glyco-FASP

As described above, crude membrane fractions of BV-2 cells were extracted using CM method 1 and CM method 2 and solubilized with strong SDS extraction buffer. After the concentration of crude membrane proteins was measured, 150 μg of proteins from each CM method was mixed 1:1 and processed by FASP. N-glycopeptides were enriched by N-Glyco-FASP, as described above.

### LC-MS/MS analysis

The peptide samples were analyzed by LC-MS on an Easy-nLC (Thermo Fisher Scientific, Odense, Denmark) that was coupled to a nanoelectrospray ion source (Thermo Fisher Scientific, Bremen, Germany) on an LTQ Velos, LTQ-Orbitrap Velos, or Q Exactive mass spectrometer (all from Thermo Fisher Scientific, Bremen, Germany). Peptides were separated on the 2-column setup with a trap column (100 μm I.D. × 3 cm) and an analytic column (75 μm ID × 15 cm) that was packed in-house with C18 resin (Magic C18-AQ 200 Å, 5 μm particles). Solvent A was 0.1% v/v formic acid and 2% acetonitrile, and solvent B was 98% acetonitrile with 0.1% v/v formic acid.

In the experiments for the crude membrane proteome, a 200-min 5% to 40% solvent B gradient was run for the initial enzyme-digested samples in MED-FASP and samples that were derived from single-FASP. A 140-min 5% to 40% solvent B gradient was applied to the second set of enzyme-digested samples in the MED-FASP procedure. In experiments on the N-glycoproteome, 3 quadruplicate runs were performed with 140 min 5% to 40% solvent B gradient. A 200-min 5% to 40% solvent B gradient was applied to the last quadruplicate run.

The spray voltage was 1.8 kV in the positive ion mode, and the temperature of the heated capillary was 325°C. Mass spectra were acquired in a data-dependent manner using a top 10 method. For low-resolution mass spectrometry on an LTQ velos, a cycle of 1 full-scan MS survey spectra (m/z 300–1800) was acquired in the profile mode. For high-resolution mass spectrometry, MS spectra were acquired on an Orbitrap analyzer with a mass range of 300–1800 m/*z* and 60,000 resolution at m/*z* 400 (Orbitrap Velos) or 300–1800 m/*z* and 70,000 resolution at m/z 200 (Q Exactive). HCD scans were acquired in Q Exactive at a resolution of 15,000. CID peptide fragments were acquired at 35 normalized collision energy (NCE) for the LTQ velos and Orbitrap velos, and HCD peptide fragments were acquired at 27 NCE.

### Data analysis for low-resolution (LR) instrument

The MS/MS spectra data from the LTQ velos were processed using the SEQUEST Sorcerer 2 platform (Sage-N Research, Milpitas, CA, USA) as described [26]. MS/MS data were searched using a target-decoy database search strategy against a composite database that contained the International Protein Index (IPI) mouse database (v3.78, 59,534 entries), and its reverse sequences were generated using Scaffold 3 (Proteome Software Inc, Portland, OR). The database search parameters were: full enzyme digest using trypsin (After KR/-) with up to 2 missed cleavages; a precursor ion mass tolerance of 1.0 Da (average mass) for glycopeptide identification; a fragment ion mass tolerance of 0.5 Da (monoisotopic mass); a static modification of 57.02 Da on Cys residues for carboxyamidomethylation; and a variable modification of 15.99 Da on Met residues for oxidation and, +2.99826 Da on Asn residues for ^18^O-deamidation. For analysis of the N-glycoproteome, the database search output results were validated using Trans-Proteome Pipeline (TPP), version 4.5 with the PeptideProphet and ProteinProphet algorithms [30].

### Data analysis for high-resolution (HR) instruments

The MS data from the LTQ Orbitrap Velos were processed in MaxQuant, version 1.2.2.5 [31] using the Andromeda search engine [32]. Precursor MS signal intensities were determined, and CID or HCD MS/MS spectra were deisotoped and filtered such that only the 6 most abundant fragments per 100 m/z range were retained. Protein groups were identified by searching the MS and MS/MS data of peptides against the IPI mouse database (v3.78, 59,534 entries), containing both forward and reversed protein sequences. For peptides that were obtained with LysC, LysC/P specificity was used. Data that were obtained from the analysis of trypsin-digested peptides were searched for trypsin/P specificity. The database search parameters were as follows: the initial precursor, CID fragment mass tolerances, HCD fragment mass tolerances were set to 7 ppm, 0.5 Da, and 20 ppm, respectively; up to 2 missed cleavages were allowed; carbamidomethylation of Cys was set as a fixed modification; oxidation of Met, acetylation of protein N-term, and, if required, ^18^O-deamidation of Asn were applied as variable modifications. Leucines were replaced by isoleucines.

All peptides, modification sites, and protein identifications were filtered at a false discovery rate (FDR) < 1%. To specify the FDR independently for peptides and proteins, peptides that belonged to proteins that did not meet the FDR threshold were removed from the dataset. Peptides were assigned to protein groups, rather than proteins. To compare protein lists between datasets, 1 representative protein of a group was defined as the lead protein, which is described in Additional file 1: Table S1 and Additional file 2: Tables S2 and S3.

### Bioinformatics analysis

Gene ontology analysis was performed using Cytoscape [33] and Plugin BiNGO 2.4 [34], the UniprotKB database [35], and the PANTHER database [36]. Pathway analysis and interaction network analysis were performed using the KEGG (Kyoto Encyclopedia of Genes and Genomes) pathways database (http://www.genome.jp/kegg), PANTHER pathway [36], and the DAVID bioinformatics tool [37]. The details of each bioinformatics tool are described in Additional file 3.

### Validation of method by western blot

To verify the crude membrane fractionation methods, control samples and crude membrane fraction samples that were prepared using the 4% SDS, KIT, and CM methods were separated by SDS-PAGE in 8% polyacrylamide gels and transferred to a PVDF membrane for western blot analysis. Details of the western blot analysis are described in Additional file 3.

## Results and discussion

### Overall experimental workflow for membrane proteome and N-glycoproteome

**Figure 1:**
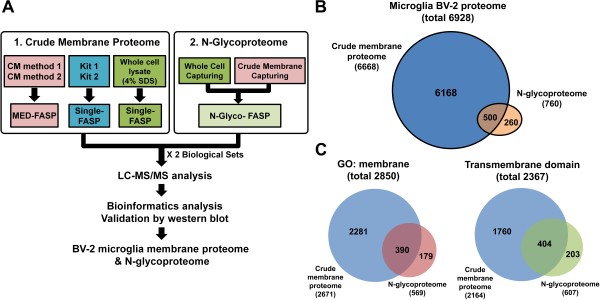
**Flowchart for analysis of crude membrane proteome and N-glycoproteome in BV-2 microglia cell line. (A)** Experiments were performed using 2 schemes. Crude membrane fractions, obtained from CM methods 1 and 2, KITs 1 and 2, and 4% SDS, were digested by MED-FASP or single-FASP. Peptides were analyzed by reverse-phase LC-MS/MS and high-resolution mass spectrometry (Orbitrap Velos and Q Exactive). To enrich N-glycopeptides, N-glyco-FASP was performed on whole-cell lysates or crude membrane fractions. **(B)** Area-proportional Venn diagram for all identified proteins with FDR < 1%. Overlap between the 2 proteomes is shown (light blue: crude membrane proteome; orange: N-glycoproteome). **(C)** Area-proportional Venn diagram for proteins identified as GO term “membrane” and transmembrane domain-containing proteins. For “GO:membrane,” the overlap between 2 proteomes is shown as a Venn diagram (light blue: crude membrane proteome; red: N-glycoproteome). For “transmembrane domain,” the overlap between the 2 proteomes is shown as a Venn diagram (light blue: crude membrane proteome; green: N-glycoproteome).

To achieve maximum coverage of the membrane proteome and N-glycoproteome in a reasonable time, we performed a novel proteomic analysis using a combination of crude membrane (CM) fractionation and protein digestion strategies without extensive peptide fractionation (Figure 1A). First, crude membrane proteins were prepared using 4 methods (CM methods 1–2 and KITs 1–2). Briefly, 200 μg of the CM fractions from CM methods 1 and 2 was digested by MED-FASP. In the first digestion, LysC and trypsin were added to the sample from CM methods 1 and 2, yielding 2 digests. In the second digestion, trypsin was then added to these digests, yielding 2 additional digested solutions. Ultimately, CM methods 1 and 2 yielded 4 fractions that were analyzed in 4 separate LC-MS/MS runs. For example, the combination of the CM fractionation method and 2-step digestions generated 4 datasets for CM method 1: MED(LysC/trypsin)_1^st^(LysC), MED(LysC/trypsin)_2^nd^(Trypsin), MED(trypsin/trypsin)_1^st^(trypsin), and MED(trypsin/trypsin)_2^nd^(trypsin).

Next, crude membranes that were extracted using commercial kits (KIT 1 and 2) were processed by single-FASP. In addition, whole-cell lysates that were processed using single-FASP were analyzed as a control set (4% SDS). Consequently, we generated 2 biological sets of a crude membrane proteome using CM methods 1 and 2 and KIT 1 and 2 and whole cell lysates (Additional file 1: Table S1). Two biological sets were analyzed using several data acquisition strategies (HR-CID and HR-HCD), based on 2 mass spectrometry platforms (Orbitrap Velos and Q Exactive, respectively). MS/MS spectra from the HR instruments were analyzed using Maxquant [31] and the Andromeda search engine [32]. Finally, the resulting data were integrated into large and heterogeneous datasets (Additional file 1: Table S1 and Additional file 2: Tables S2 and S3).

To describe the N-glycoproteome of BV-2 cells, whole-cell lysate capturing (WCC) was first processed using the N-Glyco-FASP protocol with multi-lectin enrichment and ^18^O-water [18]. To obtain a wide range of glycopeptides and improve the coverage of the N-glycoproteome, an additional analysis was performed by crude membrane fraction capturing (CMC), a method that is based on capturing N-glycopeptides from crude membrane fractions using a combination of CM methods 1 and 2 and N-glyco-FASP. Briefly, glycopeptides that were enriched from 300 μg of whole-cell lysates were analyzed on an Orbitrap Velos and Q Exactive. Also, glycopeptides that were captured from 300 μg of crude membrane fractions were analyzed on an LTQ Velos. Two biological replicates for WCC and CMC were analyzed to maximize coverage of the BV-2 N-glycoproteome. Raw files from the WCC were processed using the Maxquant-Andromeda platform, and CMC data were processed on the Sorcerer-Sequest platform. Detailed procedures of the data processing are described in Additional file 3. Detailed procedures of all experiments and an overview of the final datasets are shown in Figure 1A and Additional file 4: Figure S1.

Using all stringently filtered peptides, 6928 unique proteins were identified from 82 LC-MS/MS runs at a false discovery rate (FDR) of 1% (Figure 1B). Combining LC-MS data from the experiments for the crude membrane proteome, we identified 6668 unique proteins with a 1% FDR. In the whole-cell proteome, 3806 unique proteins were identified with a 1% FDR. Combining data from the quadruplicate analysis of N-glycosylated peptides per biological repeat, we obtained 760 glycoproteins from 1450 unique N-glycosylation sites with a 1% FDR that incorporated ^18^O-deamidated asparagine and the N-glycosylation sites of which were consistent with the canonical N!P-[S/T/rarely C] motif. As shown Figure 1C, we identified 2850 proteins that were annotated with the GO term “membrane.” Also, 2367 proteins were identified as transmembrane domain (TMD)-containing proteins in all experiments. All identification data are listed in Additional file 2: Tables S2 and S3, Additional file 5: Tables S4 nd S5, Additional file 6: Tables S6 and S7, Additional file 7: Table S8.

### General characterization of membrane proteins from BV-2 cells

**Figure 2:**
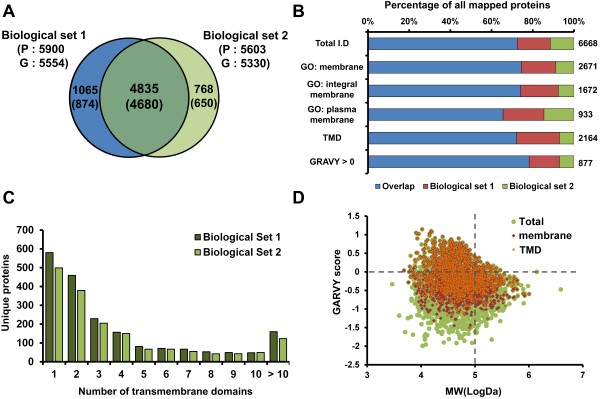
**Identification and characterization of crude membrane proteome. (A)** Overlap between proteins identified from 2 biological sets. The number of unique protein groups and the corresponding gene symbols identified from the 2 biological sets are described. The number of nonredundant genes that were identified from each biological set is shown in parentheses. **(B)** Percentage of identified proteins by functional category. All identified proteins in the 2 biological replicates were grouped into 6 categories: total identified proteins (Total I.D), proteins annotated as GO term “membrane” (GO:membrane), proteins annotated as GO term “integral to membrane” (GO:integral to membrane), proteins annotated as GO term “plasma membrane” (GO:plasma membrane), transmembrane domain (TMD)-containing proteins, and proteins with GRAVY score above 0 (GRAVY > 0). The total number of proteins identified in each category is indicated on the right of the bar. Blue bars represent percentage of proteins identified in all 2 biological sets. Red and green bars indicate percentage of proteins detected only in biological sets 1 and 2, respectively. **(C)** Distribution of number of unique proteins versus the number of predicted transmembrane domains. **(D)** Distribution of grand average hydrophobicity (GRAVY) score and molecular weight (MW) of proteins identified in the 2 biological replicates. Y-axis is the GRAVY score value, and the x-axis represents logarithmic molecular weights of the identified proteins. The GRAVY scores and log MW of 2164 transmembrane domain (TMD)-containing proteins, merged from 2 biological sets, are plotted as edged blue circles.

In our analysis of the crude membrane proteome, comprising 66 LC-MS/MS runs, 6668 protein groups were identified at an FDR of 1%. Biological sets 1 and 2 resulted in the identification of 5900 and 5603 unique protein groups, respectively (Figure 2A). Approximately 70% of identified proteins were common to all 2 biological sets. In biological sets 1 and 2, the average Andromeda identification score was 121.7 and 105.5, respectively. The absolute mass deviation ranged from 0.29 ppm to 0.60 ppm for the identified peptides in biological set 1 and 2 (Additional file 1: Table S1).

To determine the reproducibility of our analysis, correlations between protein abundance were examined in the technical replicates and biological replicates. In biological sets 1 and 2, protein abundance was calculated by summing the intensities of all peptides that were assigned to a protein. We first examined the correlation between technical replicates in each experiment. The correlation analysis of the other experiments is summarized in Additional file 4: Figure S2. Overall, the technical and biological variations in all experiments were minor (median *R* = 0.982 in technical replicates and median *R* = 0.687 in biological replicates), indicating that the crude membrane fractionation and peptide preparation methods and the mass spectrometric analysis had robust and reasonable reproducibility.

Next, we searched for the presence of specific characteristics in all identified membrane proteins. The crude membrane fractions were enriched for authentic membrane proteins. Cellular compartments of the identified proteins were analyzed using the DAVID [37], BinGO [34], and UniprotKB databases [35]. We noted that 40% to 60% of all identified proteins were *bona fide* membrane proteins, regardless of mass spectrometric method. A subsequent analysis using TMD prediction programs (SCAMPI [38], TMHMM 2.0 [39], and SOSUI [40]) suggested that 30% to 50% of identified membrane proteins contained at least 1 TMD (Additional file 4: Figure S3A).

We examined the overlap in membrane proteins and all identified proteins between the 2 biological sets (Figure 2B)—1987 membrane proteins were commonly identified as GO:membrane in the 2 biological sets, indicating that 72.5% of such proteins overlapped. Further, of 1672 proteins that were identified as integral membrane proteins, 305 (18.4%) and 128 (7.7%) appeared only in biological sets 1 and 2, respectively. Also, 1561 (72%) of 2164 TMD proteins overlapped in the 2 biological sets. Notably, the percentage of overlap in hydrophobic proteins with a GRAVY score above 0 was 78%, suggesting that crude membrane fractionation is suitable for enriching membrane proteins with high hydrophobicity.

The distribution of identified membrane proteins across the number of predicted TMDs is shown in Figure 2C. Because different informatics tools for TMD prediction have disparate outputs regarding the number and topology of the predicted TMD regions [41], several programs should be considered to provide a more comprehensive view of a membrane proteome. Thus, the representative number of predicted TMDs for each protein was defined as the highest value from SCAMPI [38], TMHMM 2.0 [39], and SOSUI [40]. Approximately 70% of all identified TMD proteins had 2 or more predicted TMDs, and 20% had 7 or more TMDs. Ten percent of TMD proteins contained 10 or more predicted TMDs. One protein (Fam38a), which had 38 predicted TMDs, was identified as Piezo-type mechanosensitive ion channel component 1 (Additional file 2: Tables S2 and S3).

We also analyzed the characteristics of our crude membrane proteome, such as protein size (MW) and hydrophobicity (GRAVY). As seen in Figure 2D, of the 6668 proteins from the 2 biological replicates, 1245 (19%) had an MW > 100 kDa and 877 (13%) had GRAVY > 0. The highest MW and GRAVY score in our proteome were 3901 kDa and 1.14, respectively. The average MW and GRAVY score of all identified proteins in the crude membrane proteome were 70.4 kDa and -0.368, respectively, versus 72.3 kDa and -0.10 in the 2379 TMD-containing proteins, respectively. Most (90%) proteins with a GRAVY score > 0 harbored TMDs, which is consistent with the high hydrophobicity of the TMD.

### Identification of BV-2 N-glycoproteome

**Figure 3:**
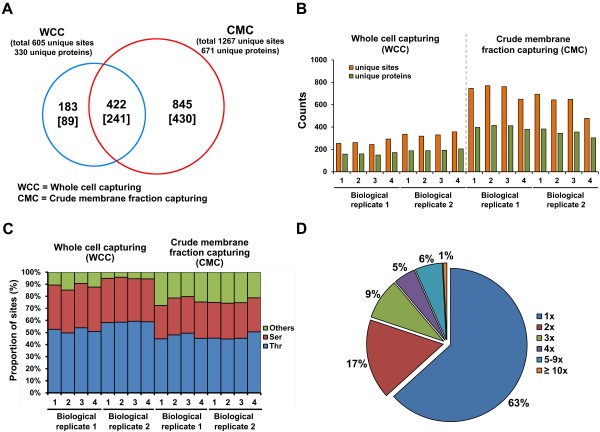
**Proteomic characterization of BV-2 N-glycoproteome. (A)** Overlap between 2 approaches (blue circle: whole cell capturing; red circle: crude membrane fraction capturing). **(B)** Identification of N-glycosylation sites and N-glycoproteins in 16 replicates by 2 different approaches. Orange bars and green bars represent the number of N-glycosylation sites and N-glycoproteins, respectively. **(C)** Frequency of consensus sequence N-x-T and N-x-S in all experiments. **(D)** Distribution of singly and multiply N-glycosylated proteins.

In analysis of BV-2 N-glycoproteome, we identified 1450 unique N-glycosites and 760 unique glycoproteins by WCC and CMC after removing the redundancy from all datasets and selecting N-glycopeptides that contained the canonical motif (Figure 3A and Table 1). We also identified 605 distinct N-glycosites for 330 unique glycoproteins by WCC and 1267 distinct N-glycosites for 671 unique glycoproteins by CMC; 422 N-glycosites from 241 glycoproteins were common in both approaches (Figure 3A and Additional file 4: Figure S4).

**Table 1:** Coverage of BV-2 N-glycoproteome by various approaches

**Experimental scheme 3**		**FDR < 1% & **^**18**^**O deamidation**			**N!P-[S/T/rarely C] motif**		
**Approach**	**Replicates**	**Redundant N-glycosites**	**Unique N-glycosites**	**Unique N-glycoproteins**	**Redundant N-glycosites**	**Unique N-glycosites**	**Unique N-glycoproteins**
WCC	Biological replicate 1	1250	545	297	1052	396	231
	Biological replicate 2	1501	576	305	1342	473	257
	Combined	2751	845	441	2394	605	330
CMC	Biological replicate 1	9536	1958	927	7380	1111	594
	Biological replicate 2	7223	1760	845	5509	1017	538
	Combined	16759	2420	1116	12889	1267	671
Total						1450	760

As shown in Additional file 4: Figure S5, the technical variation between all replicates was reasonable (overlap of 45% to 74% for unique N-glycosites and overlap of 52% to 81% for unique glycoproteins). In addition, approximately 44% of N-glycosites and 48% of glycoproteins overlapped between biological replicates by WCC. By CMC, 70% of unique N-glycosites and 69% of glycoproteins overlapped between replicates.

Although the inclusion of technical and biological replicates increased the coverage of the BV-2 N-glycoproteome, the technical and biological reproducibility ranged widely. The variability between types of mass spectrometers, differences in LC-gradient between technical replicates, and differences in individual glycopeptide preparation methods might have resulted in imperfect reproducibility between technical and biological replicates. However, in combining all replicates, the difference in the number of glycosylation sites that were identified in each replicate reflects an important advantage with regard to the number of unique identifications; thus, our experiments enhanced the coverage of the N-glycoproteome as much as possible.

As shown in Figure 3B, CMC identified significantly more N-glycosites and glycoproteins than WCC. By WCC, the quadruplicate of 2 biological replicates identified approximately 300 N-glycosylation sites, corresponding to 176 glycoproteins. In contrast, by CMC, the quadruplicate of 2 biological replicates identified an average of 670 N-glycosylation sites, corresponding to 374 glycoproteins. Because various LC-MS instruments and database processing strategies were used, a direct comparison between 2 approaches might be biased but might suggest that the wide range of approaches deepened the coverage of the BV-2 N-glycoproteome.

**Figure 4:**
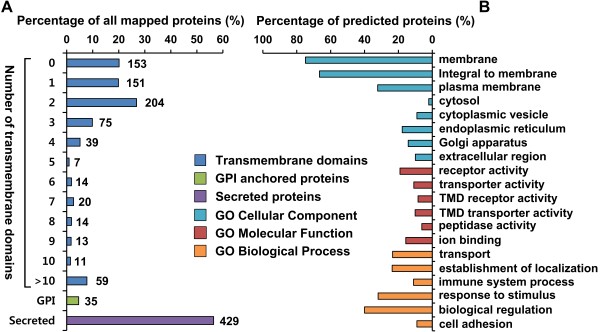
**Functional classification of identified N-glycoproteins. (A)** Analysis of transmembrane domains, GPI-anchors, and secretion of glycoproteins by multiple programs. **(B)** Gene ontology analysis of identified glycoproteins. Gene ontology of glycoproteins was analyzed using bioinformatics tools and categorized into 3 groups (cellular component, molecular function, and biological process). Membrane, integral to membrane, and plasma membrane GO terms are significantly enriched. Furthermore, the molecular function and biological process GO terms are selectively listed, with a focus on protein classes with functions associated with the sensing of stimuli and transduction of signals at the membrane.

Next, we analyzed the canonical N!P-[S/T/rarely C] motif in our BV-2 N-glycoproteome (Figure 3C). By WCC, 2394 (92%) of 2599 N-glycosylation sites matched the canonical motif without removing the redundancy. The third position of the canonical motif was occupied by Thr (612; 58.2%) or Ser (431; 41%) and Thr (830; 58.7%) or Ser (511; 36%) in biological replicates 1 and 2, respectively. Threonine occurs 1.4-fold more frequently than serine in mouse glycosylation sites [18], which is consistent with our data (1.53 = 1442/942). By CMC, 12,889 (76.0%) of 16759 N-glycosylation sites matched the canonical motif without removing the redundancy (Figure 4C). The third position of the canonical motif was occupied by Thr (4477; 47%) or Ser (2826; 30%), and Thr (3329; 46%) or Ser (2120; 29%) in biological replicates 1 and 2, respectively (Additional file 7: Table S8).

Of 760 N-glycosylated proteins, approximately two-thirds harbored a single N-glycosylation site (Figure 3D); 17% had 2 N-glycosylation sites, and 9% had 3 sites. Notably, there were 50 glycoproteins that contained 5 or more N-glycosylation sites and 6 with at least 10 sites. The highest number of N-glycosylation sites per protein was 25 for prolow-density lipoprotein receptor-related protein 1. Other glycoproteins with 10 or more N-glycosites included receptor-type tyrosine-protein phosphatase eta isoform 1, plexin B2, nicastrin, toll-like receptor 13, and lysosome-associated membrane glycoprotein 1.

### General characterization of the BV-2 N-glycoproteome

To determine the subset of proteins that was enriched by N-glyco-FASP, we examined their surface and membrane protein-specific characteristics using various bioinformatics tools. In the TMD prediction, most identified proteins had 1 or 2 TMDs (Figure 4A). In addition, 5% of proteins were predicted to contain a GPI anchor motif by GPI-SOM [42] and PredGPI [43]. TargetP [44] predicted a secretion motif in 429 (60%) of all glycoproteins, indicating that they are cleaved and secreted, despite most glycoproteins being membrane-bound (Figure 4A).

According to process of GO analysis in crude membrane proteome, we established a general GO classification for all identified glycoproteins (Figure 4B). Our GO analysis indicated that 75% of N-glycosylated proteins belonged to the category “membrane;” 66% (506 proteins) matched the category “integral to membrane;” and only 2% (16 proteins) was annotated as “cytosol” in the GO cellular compartment (GOCC) term. Moreover, 32% of the N-linked glycoproteome fell into the “plasma membrane” category, and 10% was considered “extracellular region” (Figure 4B and Additional file 7: Table S8). Considering the nonexclusive localization in GO, 42% of the N-glycoproteome lay on the outside of or beyond the plasma membrane (321 of 760 N-glycoproteins with a GO annotation). Nonsurface component categories, including the ER (18%), Golgi apparatus (12%), and cytoplasmic vesicles (9%), were overrepresented, but in nearly all cases, these annotations were nonexclusive (Additional file 7: Table S8) or validated experimentally as glycoproteins, according to the UniprotKB database.

Many molecular functions that are common in N-glycoproteins were enriched in our dataset, including receptor activity, transporter activity, TMD receptor activity, TMD transporter activity, peptidase activity, and ion binding. Transport, establishment of localization, immune function, response to stimulus, biological regulation, and cell adhesion were the predominant overrepresented biological processes (Figure 4B and Additional file 7: Table S8). Most functional categories were linked to the location of proteins at the membrane. For example, transmembrane transporter activity (*p* < 4.8×10^-10^) and cell adhesion (*p* < 3.7×10^-9^) were significantly overrepresented. In addition, many glycoproteins in our data were enriched for immunity (*p* < 5.1×10^-12^), which is a central function of microglia in the brain.

### Comparison with existing proteomics and transcriptomics data

We compared our proteome with published large-scale proteomes [12,15]. Due to the use of different species of microglia, it was difficult to compare our identified proteins with those of other studies directly. Thus, we converted the accession numbers in the database to gene names (symbols) and removed the redundancy of gene names that resulted from multiple protein isoforms in each proteome set.

As shown in Additional file 4: Figure S6A, more than two-thirds of our crude membrane proteome in microglia overlapped with 2 large-scale proteomes [12,15]. Nevertheless, approximately 1500 protein groups were identified as novel proteins in our study. Further, nearly 90% of membrane proteins from an earlier study [12] were identified as such in our data. These findings demonstrate that our proteome dataset contains many proteins that were not identified in a previous large-scale proteome analysis of cell lines.

We also compared the N-glycosylation sites in our study with the largest N-glycoproteome dataset, reported by Zielinska et al. [18]. The list of 5531 glycosylation sites from the PHOSIDA database [45] was compared directly with our N-glycoproteome (1450 sites), based on mouse IPI accession numbers (IPI_IDs). As shown in Additional file 4: Figure S6B, of our 1450 N-glycosites, 834 had with the same position, whereas 616 N-glycosylation sites were unique. Considering the IPI_IDs of glycoproteins, 453 IPI_IDs overlapped between the 2 datasets, and 307 of 760 IPI_IDs (40%) were unique to our study (Additional file 4: Figure S6B).

Our N-glycoproteome was compared with the UniProtKB database [35]. First, of 3739 mouse proteins that were annotated as glycoproteins by UniProtKB, 520 overlapped and 240 were identified as new glycoproteins in our study. Further, N-glycosylation sites were compared against UniProtKB, which included N-glycosylation information of proteins with the qualifiers “Potential,” “By similarity,” and “Experimental.” The term “Potential” indicates that there is logical or conclusive evidence, based on sequence analysis software or indirect information. When glycosylation information was obtained experimentally for other homologs and isoforms of a protein, it was tagged with the term “By similarity.”

In our study, 252 N-glycosites, corresponding to 137 glycoproteins, were identified, which has been confirmed experimentally in previous studies. A total of 740 N-glycosites, corresponding to 384 glycoproteins, were labeled as “potential” in UniProtKB. Notably, 450 N-glycosites, corresponding 349 glycoproteins, were novel N-glycosylation sites that were uncharacterized in the UniProtKB database (Additional file 7: Table S8).

Thus, we identified 556 novel N-glycosylation sites that have not been annotated in PHOSIDA or the UniprotKB database, most of which were linked to microglial function. For example, many TLR receptors, including Tlr1, Tlr2, Tlr4, Tlr7, Tlr9, and Tlr13, were identified in our crude membrane proteome. In addition, N-glycosites in Tlr1, Tlr4, Tlr7, Tlr9, and Tlr13 were identified in our N-glycoproteome. As shown in Additional file 7: Table S8, many N-glycosylation sites of Toll-like receptors in our N-glycoproteome have not been reported. We speculate that these novel sites mediate ligand recognition and regulation of TLR-mediated immune responses and signaling events.

Finally, to determine whether the N-glycoproteins were expressed predominantly in mouse microglia, we examined their expression at the transcriptome level using BioGPS [46]—705 of all identified glycoproteins were mapped in the BioGPS database [46], and the gene expression profiles for normal mouse microglia were compared with those of 96 other normal mouse tissues and cells [47,48]. Genes in microglia with 2-fold greater expression versus the median of all 96 tissues and cells were considered to be expressed specifically in microglia.

A total of 474 (67%) of 704 genes that encoded glycoproteins met the filtering criteria; 219 genes (31%) were constitutively expressed in other mouse tissues and cells. The expression of 11 genes (1.5%) was lower than in other mouse tissues and cells. The distribution of this analysis is shown in Additional file 4: Figure S7, which shows the expression levels of each gene for the 705 mapped proteins in microglia. Based on these data, microglia-specific N-glycosylation sites, particularly those that correspond to the 474 glycoproteins, are attractive candidate biomarkers and drug targets.

### Characterization of TMD-containing proteins and glycoproteins related to microglial physiology

Because membrane proteins and their glycosylation form the interface for cellular communication and interaction with the microenvironment, such as the CNS, an examination of the function of microglia in the CNS requires functional classifications to be made for such proteins. Consistent with the increasing evidence that suggests that membrane proteins and their N-glycosylation constitute a major cellular mechanism that regulates microglial function in the brain [3,5,49], we first performed literature searches and grouped the TMD-containing proteins and N-glycoproproteins in our study into functional categories using the PANTHER protein class ontology database [36] (Additional file 4: Figures S8A-C).

Briefly, we performed literature searches to ensure that our BV-2 proteome as examined as markers for microglia and had functional links to microglial physiology (Table 2). Nearly all known markers that are used to discriminate microglia from other CNS-resident cells and monocytes and macrophages were identified in our study. Also, several N-glycosylation sites in microglia markers were identified in our N-glycoproteome, allowing us to distinguish microglia from other macrophages and monocytes in the CNS. Moreover, several significant membrane proteins in microglial function were identified in the membrane proteome and N-glycoproteome (Table 2). The detailed literature search and functional categories are described in Additional file 3. Also, a detailed list of functional classes for TMD-containing proteins and N-glycoproteins is provided in Additional file 8: Table S9.

**Table 2:** List of membrane proteins and N-glycosylation sites associated with microglial physiology

**Group**	**Protein type**	**Representing name**	**Gene symbol**	**Protein name**	N-glycosites (*Noble site)	**Max. sequence coverage**
**Microglia marker**		CD11b	Itgam	Integrin alpha-	58 N, 391 N, 696 N, 734 N, 907 N, 941 N, 1022 N, 1045 N	13.2% (B1)
		CD18	Itgb2	Integrin beta-2	51 N, 502 N, 626 N, 644 N	29.8% (B2)
		CD11c	Itgax	Integrin alpha-X	393 N*	Glycol-only
			Ptprc	Receptor-type tyrosine-protein phosphatase C	210 N*, 215 N*, 247 N*, 268 N*, 279 N*, 290 N*, 304 N*, 311 N*, 322 N*, 347 N*, 384 N*, 427 N*, 446 N*, 489 N*, 1237 N*	27.9% (B2)
		CD68	Cd68	Macrosialin	129 N*, 134 N*, 169 N*, 178 N, 260 N	16.1% (B2)
		F4/80 antigen	Emr1	EGF-like module-containing mucin-like hormone receptor-like 1	405 N*, 413 N*, 417 N*, 498 N	21.3% (B2)
		Iba1	Aif1	Allograft inflammatory factor 1	-	8.2% (B2)
**Ion channel**	Calcium channel	TRPs	Trpm7	Transient receptor potential cation channel subfamily M member 7	-	1.7% (B1)
			Trpc2	Short transient receptor potential channel 2	-	0.8% (B1)
			Trpc4	Short transient receptor potential channel 4	-	0.8%(B1)
			Trpv2	Transient receptor potential cation channel subfamily V member 2	567 N*	33.1%(B1)
	Potassium channel	Inward rectifier potassium channels	Kcnj2	Inward rectifier K(+) channel Kir2.1	-	3.3%(B1)
		BK channels	Kcnma1	Calcium-activated potassium channel subunit alpha-1	-	1.6%(B2)
			Kcnu1	Calcium-activated potassium channel subunit alpha-3	339 N*	Glyco-only
			Kcnmb3	Calcium-activated potassium channel subunit beta-3	-	4.6%(B2)
			Kcnab2	Voltage-gated potassium channel subunit beta-2	-	8.6%(B2)
	anion channels	CLIC-1 chloride channels	Clic1	Chloride intracellular channel protein 1	-	60.6%(B2)
	other channels	Proton channels	Hvcn1	Hydrogen voltage-gated channel 1	-	13.4%(B1)
**Neuro-Trans-mitter Receptor**	Purino ceptors	P2X4	P2rx4	P2X purinoceptor 4	75 N, 131 N, 153 N*, 184 N, 208 N	21.6%(B2)
		P2X7	P2rx7	P2X purinoceptor 7	74 N, 187 N, 202 N, 213 N	12.8%(B1)
		P2Y6, P2Y10	P2ry6	P2Y purinoceptor 6	-	3.7%(B1)
			A630033H20Rik	Novel protein similar to purinergic receptor P2Y G-protein coupled 10 (P2ry10)	-	3.7%(B1)
	Glutamate receptor	AMPA receptors	Gria2	AMPA-selective glutamate receptor 2	-	1.7%(B2)
		Metabotropic glutamate receptors	Grm1	Isoform 1 of Metabotropic glutamate receptor 1	-	2%(B1)
		GABA receptors	Gabra5	Gamma-aminobutyric acid receptor subunit alpha-5	-	1.7%(B1)
**Neuro-hormones and neuro-modu-lators receptor**	Glucocorticoid receptors	Glucocorticoid receptor	Nr3c1	Glucocorticoid receptor	-	4.2%(B1)
	Opioid receptors		Sigmar1	Sigma 1-type opioid receptor	-	43.5%(B2)
			Ogfr	Opioid growth factor receptor	-	17.1%(B2)
	Cytokine receptors	TNF-alpha receptors	Tnfr2	TNF-alpha receptor 2	-	9.5%(B1)
			Tnfrsf1b	TNF receptor superfamily member 1B	-	9.5%(B1)
			Tnfrsf26	TNF receptor superfamily member 26	136 N*	14.2%(B1)
		Interleukin receptors	Il2rg	Interleukin 2 receptor, gamma chain	96 N, 159 N*, 164 N*, 306 N*	5.1%(B1)
			Il10rb	Interleukin-10 receptor subunit 2	51 N*	11.4%(B1)
			Il13ra1	Interleukin-13 receptor subunit alpha-1	262 N	Glyco-only
			Il6st	Interleukin-6 receptor subunit beta	43 N	Glyco-only
			Il10ra	Interleukin-10 receptor subunit alpha	113 N*	Glyco-only
			Il4ra	Isoform 1 of Interleukin-4 receptor subunit alpha	163 N	Glyco-only
			Csf2rb	Cytokine receptor common subunit beta	141 N*	2.7%(B1)
**Other receptor system**		Notch receptors	Notch2	neurogenic locus notch homolog protein 2	-	3.3%(B1)
		Complement receptors	C5ar1	Complement component 5a receptor 1	-	9.4%(B1)
		Macrophase colony-stimulating factor receptors	Csf1r	Macrophage colony-stimulating factor 1 receptor	-	16.6%(B1)
			Csf2ra	Granulocyte-macrophage colony-stimulating factor receptor subunit alpha	132 N*, 165 N, 269 N*	Glyco-only
		Formyl peptide receptors	Fpr1	fMet-Leu-Phe receptor	-	9.3%(B2)
		CD200 receptors	Cd200r1	CD200 cell surface glycoprotein receptor	93 N, 192 N*	5.5%(B1)
			Cd200r4	CD200 cell surface glycoprotein receptor-like 4	-	12.6%(B1)

### Pathway analysis of BV-2 crude membrane proteome and N-glycoproteome

**Figure 5:**
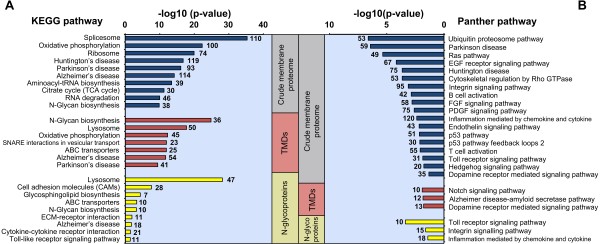
**Pathway enrichment analysis for identified membrane proteins, transmembrane-containing proteins, and N-glycoproteins.** Pathway enrichment analysis of 3 clusters—crude membrane proteome, transmembrane domains (TMDs), and N-glycoproteins—using the KEGG pathway database **(A)** and Panther pathway database **(B)**. Logarithmic corrected p-values for significant overrepresentation are shown. The number of proteins in each pathway is indicated to the right and left of the bars. Each cluster is indicated by a colored box.

To examine the pathways of the molecular interactions and reaction networks in our BV-2 membrane proteome and N-glycoproteome, we analyzed our data using the KEGG pathways database (Figure 5A). In 6668 proteins that were enriched in the crude membrane proteome, the predominant cellular pathways were RNA biogenesis, protein metabolism, and citric acid (TCA) cycle; neurodegenerative diseases also appeared, such as Huntington, Parkinson, and Alzheimer diseases. For TMD-containing proteins that were enriched in crude membrane fractions, the chief membrane-associated pathways were N-glycan biosynthesis, lysosome, ABC transporters, and SNARE interactions in vesicular transport. Further, N-glycosylated proteins were enriched in many pathways that are linked to the plasma membrane, such as cell adhesion molecules (CAMs), ECM-receptor interactions, cytokine-cytokine receptor interactions, and Toll-like receptor signaling (Additional file 8: Table S10).

An additional pathway analysis was performed using the PANTHER database [36] to study signaling pathways (Figure 5B and Additional file 8: Table S11). The crude membrane proteome was significantly enriched in many pathways in neurodegenerative diseases and microglia-mediated inflammation (Figure 5B). Detailed information on these signaling pathways is described in Additional file 3. We also detected N-glycoproteins that are associated with microglia-associated immune responses. For example, we noted many N-glycosites on proteins that are involved in Toll receptor signaling, integrin signaling, and chemokine- and cytokine-mediated inflammation (Figure 5B). In particular, Toll receptor signaling is discussed in Additional file 3. Because N-glycosylation is involved in many processes that are associated with microglial function in the CNS, such as cell-cell and receptor-ligand interactions and immune responses, we hypothesize that N-glycosylation mediates microglia-induced innate immunity [49].

### Multiplexed proteomic CD antigen phenotyping based on membrane proteins and N-glycosylation

Cluster of differentiation (CD) antigens are cell surface molecules that are used to immunophenotype cells [50]. Because much disease pathogenesis and progression involve immune system activation or suppression, these antigens are a unique tool to monitor host responses [51]. Further, multiplexed phenotyping that involves parallel measurements of CD antigens can help identify expression pattern signatures that are associated with specific disease states [51].

**Figure 6:**
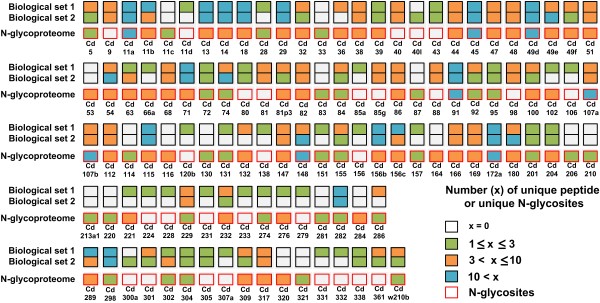
**Extensive CD antigen phenotyping of BV-2 cells.** Overview of 114 CD antigens identified in BV-2 cells by crude membrane fractionation and N-glycopeptide capture. The inner color code of the squares denotes the number of assigned unique peptides per CD antigen for 2 biological replicates of the crude membrane proteome. Also, the inner color code of the red squares denotes the number of unique N-glycosylation sites per CD antigen for the N-glycoproteome in BV-2 cells.

Multiplexed CD phenotyping of immune cells has traditionally depended on well-characterized monoclonal antibodies. However, antibody-based approaches are commonly restricted to the few existing antibodies. Thus, we performed multiplexed proteomic CD antigen phenotyping using data from the MS-based membrane proteome and N-glycoproteome; 114 CD proteins were expressed under normal conditions in BV-2 cells (Figure 6).

Sixty-two CDs were identified with greater than 3 unique peptides per CD in at least 1 biological set, whereas 16 proteins were identified by N-glycosylated peptides. Notably, 78% (89 proteins) of CD antigens were glycosylated, and 54% (61 proteins) was multiply glycosylated, demonstrating the robustness of cell surface phenotyping with our proteomics approaches—ie, crude membrane fractionation and enrichment of N-glycosylation sites. The identified CD antigens included well-known microglia surface markers, such as CD11b, CD11c, CD45, and CD68 [5]. Moreover, CD169 (sialoadhesion), CD204 (MSR), and CD206 (mannose receptor), which are targets for recognizing macrophages and macrophage-like cells, were identified. Many CD antigens were highly expressed, including CD14, CD36, CD39, CD40, CD45, CD47, CD54, and CD106, which are linked to microglial activation and microglial functions in immune responses and neurodegenerative diseases.

Consequently, our data confirmed the expression of 114 CD antigens in microglia experimentally, which can be used to select and evaluate antibodies in microglial functional studies. In addition to Antibody-based applications, our data allow one to choose fragment ions of peptides and glycopeptides for MS workflows by peptide-targeted selected reaction monitoring (SRM) assay [52]. The combination of crude membrane fractionation and N-glycoprotein enrichment with quantitative SRM assays will contribute significantly to the comprehensive and systematic validation of changes in the abundance of targeted cell surface proteins. Also, such approaches that enable one the systematically compare cell surface phenotypes under various conditions have the potential to improve the classification of and examine surface proteins that have clinical interest in neurodegenerative diseases.

### Validation of enrichments of BV-2 crude membrane proteome and N-glycoproteome by western blot analysis

**Figure 7:**
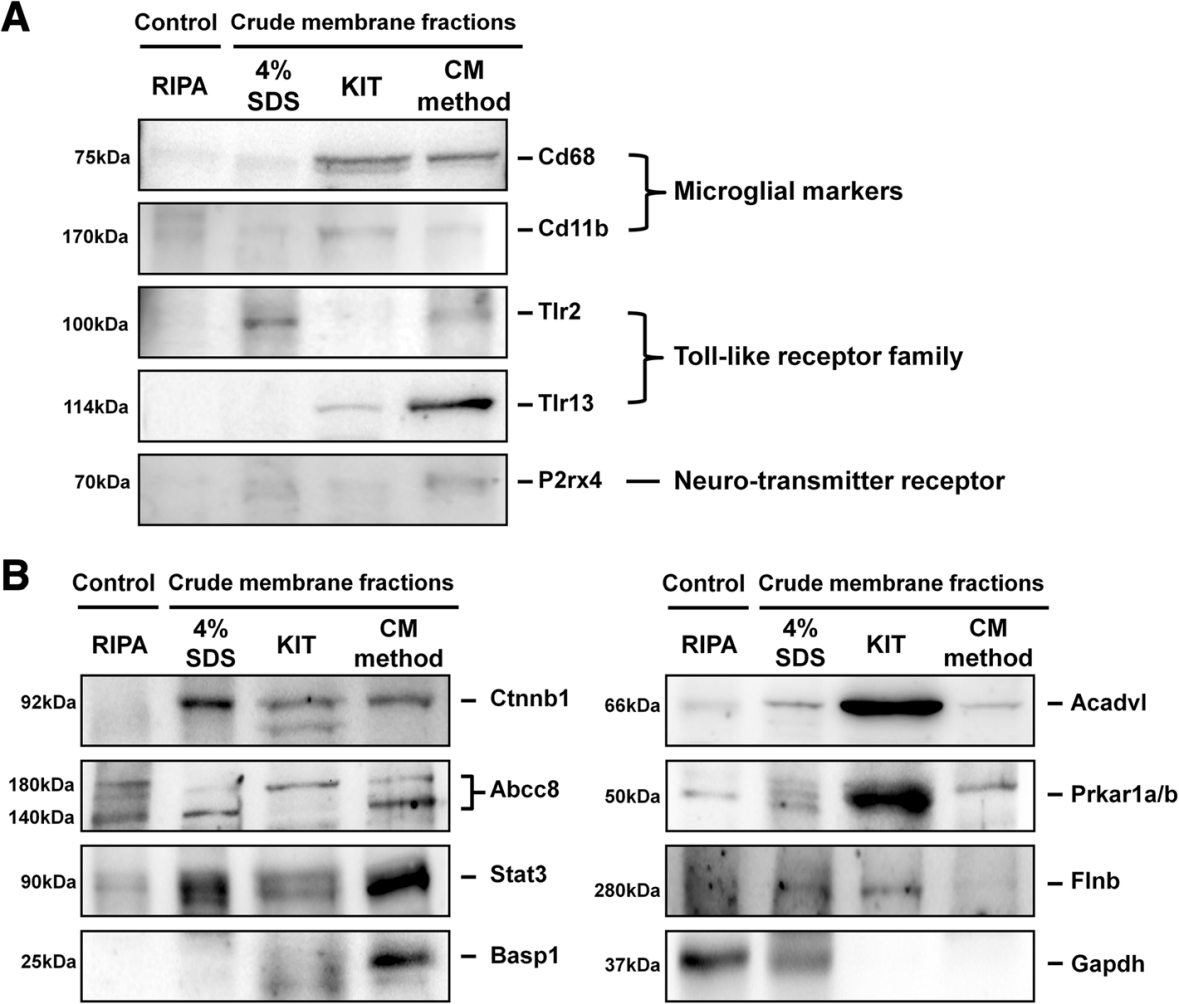
**Western blot analysis of membrane proteins and N-glycosylated proteins.** The abundance of 5 proteins that are closely associated with microglial functions **(A)** and 7 membrane and N-glycosylated proteins and 1 cytosolic protein **(B)** was measured by western blot in control (RIPA) and crude membrane fraction samples.

Using antibodies and western blot analysis, the major proteins that were identified in our crude membrane proteome and N-glycoproteome were validated. The abundance of 5 major proteins (Cd11b, Cd68, Tlr2, Tlr13, and P2rx4) that were closely associated with microglial function increased after crude membrane fractionation (Figure 7A). Further, several membrane proteins and N-glycosylated proteins (Ctnnb1, Abcc8, Stat3, Basp1, Acadvl, Prkar1a/b, and Flnb) were detected in the crude membrane-enriched fractions. In addition, the cytosolic protein (Gapdh) was used as a control for crude membrane fractionation (Figure 7B). Collectively, these data suggest that our crude membrane fractionation strategies are useful methods for studying membrane proteins and N-glycosylated proteins in microglia.

## Conclusion

We performed large-scale analyses of membrane proteins and N-linked glycopeptides from the BV-2 microglia cell line. Without extensive peptide fractionation or MUDPIT analysis, our combination of sample preparation methods—crude membrane fractionation, FASP-based peptide preparation, glycopeptide enrichment using N-glyco-FASP, and integration of heterogeneous datasets at a high accuracy level—allowed us to identify 2850 membrane proteins and 1450 unique N-glycosylation sites on 760 glycoproteins, resulting in the identification of 6928 protein groups in BV-2 cells.

Our study is the most comprehensive analysis of the membrane proteome and N-glycoproteome in microglia, providing a rich resource that can be used to examine the functions of membrane proteins and their N-linked glycosylation with regard to microglial activities in the brain, including microglial activation, cell-to-cell communication, innate immune responses, and inflammatory activity. Further, information on novel N-glycosylation sites and N-glycosylation sites that are involved in microglial immune responses can be used by ongoing clinical studies on the membrane proteome or N-glycoproteome to target microglial proteins that mediate the pathology of neurological diseases.

## Abbreviations

CNS: Central nervous system; AD: Alzheimer disease; PD: Parkinson disease; MS: Mass spectrometry; PTM: Posttranslational modification; FASP: Filter-aided sample preparation; MED-FASP: Multienzyme digestion-FASP; SDS: Sodium dodecyl sulfate; LC-MS/MS: liquid chromatography-coupled tandem mass spectrometry; TPP: Trans-Proteome Pipeline; HR: High-resolution; LR: Low-resolution; FDR: False discovery rate; CID: Collision-induced dissociation; HCD: Higher-energy collisional dissociation; GO: Gene ontology; KEGG: Kyoto Encyclopedia of Genes and Genomes; IPI: International Protein Index; WCC: whole-cell capturing; CMC: crude membrane capturing; PSM: Peptide spectrum matche; TMD: Transmembrane domain; GRAVY: Grand average of hydropathicity index; MW: Molecular weight; CD: Cluster of differentiation.

## Competing interests

The authors declare that they have no competing interests.

## Authors’ contributions

DH and SM conceived the project, conducted all experiments including sample preparation, LC-MS/MS, data analysis, and bioinformatics analysis, and drafted he manuscript. YK, HM, and JW participated in mass spectrometry experiments and data analysis. YK directed the project and critically revised the manuscript. All authors read and approved the final manuscript.

## Supplementary Material

Additional file 1: Table S1 Information on peptide and protein identificaiton for crude membrane proteome. Click here for file

Additional file 2: Table S2 Total protein list identified in biological set 1. **Table S3.** Total protein list identified in biological set 2. Click here for file

Additional file 3 Supplementary text. Click here for file

Additional file 4: Figure S1 Detailed flowchart for the identification of membrane proteins and N-glycoproteins. **Figure S2.** Technical and biological reproducibility in experiments for crude membrane proteome. **Figure S3.** Complementarity of multiple strategies for comprehensive coverage of crude membrane proteome. **Figure S4.** Comparison between biological replicates by WCC and CMC for the N-glycoproteome. **Figure S5.** Technical reproducibility and biological reproducibility in experiments for N-glycoproteome profiling. **Figure S6.** Comparison of BV-2 crude membrane proteome and N-glycoproteome. **Figure S7.** Comparison with transcriptomics data to identify microglia-specific glycoproteins. **Figure S8.** Characterization of TMD-containing proteins and N-glycoproteins. **Figure S9.** Detailed information on Toll-like receptor (TLR) family. Click here for file

Additional file 5: Table S4 N-glycoproteome in WCC at FDR < 1%. **Table S5.** N-glycosylation sites containing N!P-[S/T/rarely C] motif by WCC. Click here for file

Additional file 6: Table S6 N-glycoproteome by CMC at FDR < 1%. **Table S7.** N-glycosylated peptides containing N!P-[S/T/rarely C] motif by CMC. Click here for file

Additional file 7: Table S8 Unique N-glycosylation site. Click here for file

Additional file 8: Table S9 PANTHER molecular function analysis. **Table S10.** KEGG pathway analysis. **Table S11.** PANTHER pathway analysis. Click here for file
