# Supplementary material for: Characterization of the membrane proteome and N-glycoproteome in BV-2 mouse microglia by liquid chromatography-tandem mass spectrometry
Source: BMC Genomics. 2014 Feb 4;15:95. doi: 10.1186/1471-2164-15-95 (PMC3938046; doi:10.1186/1471-2164-15-95)
Supplement: Additional file 3 — Supplementary text. [file 1471-2164-15-95-S3.docx]

**Supplementary Text**

**Supplementary Methods**

***Bioinformatics analysis***

Gene ontology (GO) analysis was performed using DAVID Bioinformatics resources 6.7 tools[[1](#_ENREF_1)], the UniprotKB database[[2](#_ENREF_2), [3](#_ENREF_3)], Cytoscape[[4](#_ENREF_4)], and BiNGO 2.4[[5](#_ENREF_5)]. First, GO annotations of leading proteins in each group were extracted from DAVID tools and the UniprotKB database using IPI accession numbers and Uniprot accession numbers, respectively. Protein groups that had no GO classifications from DAVID tools or the UniprotKB database were subjected to BiNGO analysis using Cytoscape. Prior to the BiNGO analysis, the hypergeometric statistical test, Benjamini & Hochberg false discovery rate correction, multiple test correction, and *Mus musculus proteome* were set as the analysis parameters. BiNGO provides *P-value* statistics, based on the probability of the occurrence of genes/proteins in the defined ontological categories. For our analysis, the level of significance was set to *P*-value < 0.05. For our membrane proteome and N-glycoproteome, cellular components, molecular functions, and biological processes were analyzed separately. Consequently, the GO annotations of each protein group that were obtained using multiple tools were merged in Supplementary Tables S2-3.

Pathway analysis was performed using the KEGG (Kyoto Encyclopedia of Genes and Genomes) pathways database (http://www.genome.jp/kegg), PANTHER pathway database[[6](#_ENREF_6)], and DAVID bioinformatics tools[[1](#_ENREF_1)]. DAVID tools provided the corresponding information on *P*-value, count, percentage (%), and fold-enrichment for each KEGG and PANTHER pathway. The *P*-value was EASE score, a modified Fisher exact *P*-value, which reflects the probability that the uploaded protein list is associated with a specific KEGG and PANTHER pathway by random chance [[7](#_ENREF_7)]. KEGG and PANTHER pathways with a *P*-value ≤ 0.05 were considered significantly enriched pathways.

GPI-SOM[[8](#_ENREF_8)] and pred-GPI[[9](#_ENREF_9)] were used to predict GPI-anchoring signal sequences. By GPI-SOM analysis, proteins that contained both C- and N-terminal signal sequences were only accepted as GPI-anchored proteins. In the pred-GPI analysis, proteins with a highly probable or probable prediction accuracy (above 99.5% specificity) were only selected as GPI-anchored proteins. The TargetP 1.1 server [[10](#_ENREF_10)] was used to predict secretory proteins among N-glycoproteins. Analysis was performed after setting for nonplant networks.

***Validation of method by western blot***

To validate the crude membrane fractionation methods, control samples and crude membrane fraction samples were prepared using the 4% SDS, KIT, and CM methods. Control samples were prepared in RIPA buffer (50 mM Tris-HCl pH 7.4, 1 mM EDTA, 150 mM NaCl, 10 mM sodium pyrophosphate, 10 mM NaF, 0.5% (v/v) NP-40, 1 mM PMSF, and a protease inhibitor cocktail). Samples, containing 10 μg of protein, were separated by SDS-PAGE on 8% polyacrylamide gels and transferred to a PVDF membrane for western blot analysis. After being incubated for 2 hr with blocking solution [5% (w/v) BSA in TBS-T (0.05% (v/v) Tween 20 in Tris-buffered saline], the membrane was probed with the primary antibodies overnight at 4°C. After being washed 5 times with TBS-T, the membrane was incubated with horseradish peroxidase (HRP)-conjugated secondary antibody to mouse, goat, or rabbit (Santa Cruz Biotechnology, CA, USA), diluted in blocking solution (final concentration, 1:5000 μg/mL) for 2 hr at room temperature. After every incubation step, the membranes were washed 5 times for 10 min each in TBS-T.

Bands were developed with the ECL Plus western blot kit (GE Healthcare, Piscataway, NJ, USA). Western blots were visualized on an LAS-4000 mini luminescent image analyzer (Fuji film, Tokyo, Japan). The following primary antibodies were used: Integrin αM (Santa Cruz Biotechnology, sc-6614, 1:1000), CD68 (Santa Cruz Biotechnology, sc-9139, 1:1000), P2X4 (Santa Cruz Biotechnology, sc-28764, 1:1000), TLR2 (Santa Cruz Biotechnology, sc-10739, 1:500), TLR13 (Pierce, PA5-23107, 1:500), PKA Iα/β (Santa Cruz Biotechnology, sc-28893, 1:1000), STAT3 (Santa Cruz Biotechnology, sc-482, 1:1000), ACADVL (Santa Cruz Biotechnology, sc-376239, 1:1000), NAP-22 (Santa Cruz Biotechnology, sc-32837, 1:1000), filamin 3 (Santa Cruz Biotechnology, sc-376241, 1:1000), β-catenin (Santa Cruz Biotechnology, sc-7963, 1:1000), ABCC8 (Santa Cruz Biotechnology, sc-5789, 1:1000), and GAPDH (Santa Cruz Biotechnology, sc-25778, 1:1000).

**Supplementary Results & Discussion**

***Processing of N-glycoproteome data***

For the WCC approach, the data were analyzed with MaxQuant, [[11](#_ENREF_11)] specifying a false discovery rate of 1% at the peptide and modification site level. We identified 2751 redundant N-glycosites, corresponding to 440 unique glycoproteins, at an FDR < 1% (Table 1 and Supplementary Table S4-S5). Of 2751 N-glycosites, only N-glycosites with a localization probability of at least 0.75 were analyzed further, resulting in 2599 redundant N-glycosites. After a filtering step, the average localization probability of all identified sites was 0.991. Also, the average Andromeda score was 107.5, indicating that the peptide identification and localization of the modification in the peptide sequence at single-amino-acid resolution were unambiguous.

For the CMC approach, all MS/MS spectra from the LTQ Velos were searched using the Sorcerer-Sequest platform. First, spectra that contained at least 1 ^18^O-deamidated asparagine were selected to remove nonglycosylated peptides. The results were also filtered using the PeptideProphet probability score [[12](#_ENREF_12)] to establish N-glycopeptide datasets at an FDR < 1.0 %. An average PeptideProphet probability score of 0.92 was set as the threshold in all 8 technical replicates. Overall, we identified 14,121 redundant N-glycopeptides, corresponding to 1116 unique glycoproteins, at an FDR level < 1.0% (Supplementary Table S6-S7). Xcorr and deltaCN values of all identified N-glycopeptide with an FDR < 1% were 3.59 and 0.337, respectively. Also, the average PeptideProphet probability score of N-glycopeptides that were identified at an FDR < 1% was greater than 0.99, indicating the reliability of N-glycopeptide identification at the peptide level with an FDR < 1%.

Whereas FDR estimation at the modification site level and localization probability were used to assess modification sites in the WCC, data for CMC were processed at the peptide level, based on PeptideProphet probability using the Sorcerer-Sequest platform. Also, all CMC data were collected by low-resolution (LR) mass spectrometry (LTQ velos). These differences could have led to the improper localization of modification sites. To remove this ambiguity, we processed the data differently than for the WCC.

First, a SEQUEST database search was carried out with a 1 Da precursor ion error tolerance to separate mass differences in ^18^O-deamidation (2.99 Da) accurately. Next, N-glycosylation motif information and accurate mass binning were used to validate peptides using PeptideProphet [[12](#_ENREF_12)]. Then, the FDRs of all MS/MS spectra were estimated with the threshold of PeptideProphet probability that was calculated from the mayu module of Trans-Proteomic Pipeline (TPP), not with manually calculated thresholds. Finally, peptides that contained the canonical motif (N-!P-[S/T/rarely C]) were selected. Although the enrichment yield (57%) of the canonical motif (N-!P-[S/T/rarely C]), based on unique N-glycosites, was lower than that of N-glycopeptides (76.9%), the average SEQUSET scores (XCorr, deltaCN, and Spscore) of the final data were 3.59, 0.346, and 875, respectively. Also, the average PeptideProphet probability score and average percentage of matched experimental MS/MS fragments among the total theoretical fragment ions were 0.992 and 53.57%, indicating the reliability of N-glycopeptide identification by CMC.

***Crude membrane fractionation and two-step digestion increase the depth of the membrane proteome***

Due to the low solubility of membrane proteins, their resistance to proteolysis, and poorly resolution of protein separation, the comprehensive analysis of membrane proteins has faced many technical challenges[[13](#_ENREF_13), [14](#_ENREF_14)], leading to inefficient digestion and decreased recovery of peptides, limiting the precision and confidence of protein identification[[13](#_ENREF_13), [14](#_ENREF_14)]. To overcome these hurdles and enhance the profiling coverage, we used a multiplex strategy as described above, comprising (1) 4 crude membrane fractionation methods, (2) multiple enzyme digestion, based on FASP (MED-FASP), and (3) inclusion of technical and biological replicates.

The competition of crude membrane fractionations was evaluated by comparing our fractionation methods with commercial kits and non-fractionation protocols. Thus, the results of our multiplex strategy with regard to membrane proteome coverage were estimated, based on the percentage of proteins that were identified as membrane proteins based on GO classification, and the prediction of TMDs in our data. Supplementary Tables S1-S3 shows the significant parameters for all experiments, such as the number of proteins that were annotated as membrane protein and the percentage of coverage.

As shown in Supplementary Figure S3A and S3B, most identification was made with a combination of CM method 1 and MED_FASP (LysC/trypsin) in all biological sets. In 2 biological sets, CM method_1 and MED_FASP (LysC/trypsin) identified 3 times as many membrane proteins as in the whole-cell lysate control set (Supplementary Figure S3A and S3B). Particularly with regard to TMD-containing proteins, CM method 1 and MED_FASP (LysC/trypsin) identified 5 times as many proteins versus the control set. In addition, CM method 2 and MED_FASP (LysC/trypsin) identified twice as many membrane proteins over the control set. However, the combination of CM methods 1 and 2 and MED_FASP (trypsin/trypsin) identified a similar number of proteins versus the commercial kits (Supplementary Figure S3B).

The Venn diagrams in Supplementary Figure S3C show the overlap in membrane proteins and TMD-containing proteins between the 4 fractionation methods (CM method 1, CM method 2, KIT1, and KIT2), which were digested by single-FASP in all biological sets. In each biological set, 38% to 49% of membrane proteins overlapped, versus 37% to 53% of TMD-containing proteins, between the 4 fractionation methods, indicating that these methods provide complementary coverage and that their combination effects comprehensive coverage of the membrane proteome.

**Characterization of TMD-containing proteins and glycoproteins related to microglial physiology**

We sorted TMD-containing proteins and N-glycoproproteins into functional categories using a literature search and the PANTHER protein class ontology database [[6](#_ENREF_6)] (Supplementary Figure S8). A detailed list of functional protein classes of the TMD proteins and N-glycoproteins is provided in Table 2 and Supplementary Table S9.

Many microglial markers and their N-glycosylation sites were identified in our study, including CD11b, CD18, CD11c, CD34, CD45, CD68, F4/80 antigen, and Iba1. Also, several N-glycosylation sites in CD11b, CD18, CD11c, CD68, and F4/80 antigen were identified from the N-glycoproteome. Although the exact function and structure of glycosylation are unknown[[15](#_ENREF_15)], lectin staining was used to identify microglia and traditional microglial markers, such as CD11b, CD18, and Iba1. Moreover, several membrane proteins that are significant in microglial function were identified in the membrane proteome and N-glycoproteome and categorized into 5 groups: ion channel, neurotransmitter receptor, neurohormone and neuromodulator receptors, TLRs, and other receptor systems. Fifty-three membrane proteins and 98 N-glycosites were included in protein groups that are linked to microglial functions in the brain (Table 2 and Supplementary Table S9).

In a functional catalog, based on the PANTHER database, 1617 of 2579 TMD-containing proteins were functionally annotated and sorted by *P*-value. The top 10 categories were identified: Transporter (*P* < 1.41x10^-79^), Other transporter (*P* < 3.9x10^-36^), Glycosyltransferase (*P* < 2.21x10^-20^), Cation transporter (*P* < 9.44x10^-18^), Membrane traffic protein (*P* < 1.68x10^-12^), SNARE protein (*P* < 3.7x10^-12^), Transferase (*P* < 9.2x10^-11^), ABC transporter (*P* < 5.03x10^-10^), Other receptor (*P* < 8.41x10^-9^), and Oxidoreductase (*P* < 3.86x10^-8^) (Supplementary Figure S8A).

In addition, N-glycosylated proteins were functionally grouped into top 10 categories, ranked by *P*-value: other receptor (*P* < 1.06x10^-15^), Glycosyltransferase (*P* < 3.24x10^-13^), Transporter (*P* < 8.16x10^-10^), Glycosidase (*P* < 1.28x10^-9^), Cytokine receptor (*P* < 9.01x10^-7^), Hydrolase (*P* < 1.16x10^-6^), Receptor (*P* < 1.36x10^-5^), Cell adhesion molecule (*P* < 1.36x10^-5^), Ig receptor family member (*P* < 1.66x10^-5^), and other cell adhesion molecule (*P* < 2.31x10^-5^) (Supplementary Figure S8B).

Finally, the BV-2 membrane proteome and N-glycoproteome formed 7 major functional protein classes, according to our functional classification and literature search (Supplementary Figure S8C). The receptor and transporter groups made a larger contribution to the BV-2 membrane proteome, based on the numbers of proteins that were in these categories. The receptor group consists primarily of transmembrane receptors, TNF receptors, Ig receptors, and Toll-like receptors, indicating their significance in microglia, as previously described[[15](#_ENREF_15)]. The representative proteins for each functional group were the interleukin receptor family, interferon receptor family for cytokine receptors, ADAM family for proteases, integrin family, CAM family for cell adhesion molecules, ABC transporter family, MDR transporter family, MRP transporter family for transporters, TRP family, BK channels, CLIC1 for ion channels, and beta-1,4-GalT family for proteases.

**Detailed pathway information on the BV-2 membrane proteome**

Various signaling pathways that are related to microglial function were enriched in our analysis using the PANTHER pathway database (Supplementary table S11). For example, signaling pathways that are mediated by growth factors, such as EGF, FGF, and PDGF, have significant functions in microglial inflammatory responses[[16](#_ENREF_16), [17](#_ENREF_17)]. Moreover, many proteins were included in microglial activation and innate immune responses, including the integrin signaling pathway[[18](#_ENREF_18)], chemokine and cytokine signaling pathway[[19](#_ENREF_19)], cytoskeletal regulation by Rho GTPase[[20](#_ENREF_20)], Toll-like receptor signaling pathway[[21](#_ENREF_21), [22](#_ENREF_22)], dopamine receptor-mediated signaling pathway [[23](#_ENREF_23), [24](#_ENREF_24)], and endothelin signaling pathway [[25](#_ENREF_25)].

TMD-containing proteins were significantly enriched in 3 signaling pathways: Notch signaling, Alzheimer disease-amyloid secretase, and dopamine receptor-mediated signaling. We noted 9 TMD-containing proteins that mediate Notch signaling: Aph1a, Aph1c, Adam10, Adam17, Ncstn, Notch2, Bptf, Rab30, and Psen2. Although Notch signaling mediates development in the brain, recent studies have suggested that it modulates the activation of microglial cells and microglia-mediated inflammatory responses[[26-28](#_ENREF_26)].

Further, Adam9, Adam10, Adam17, Mapkapk2, Aph1a, Aph1c, App, Psen1, Psenen, Ncstn, and Cacnald were annotated with the amyloid secretase pathway in Alzheimer disease (Supplementary Table S11). Adam9, Adam10, and Adam17 possess alpha-secretase activity [[29](#_ENREF_29)], whereas Aph1, Psen1, Psenen, and Ncstn form the gamma-secretase complex [[30](#_ENREF_30), [31](#_ENREF_31)]. Three proteases—alpha-secretase, beta-secretase, and gamma-secretase—process amyloid precursor proteins [[32](#_ENREF_32)]. Unlike beta-secretase (BACE), which produces amyloid-beta that is assembled into senile plaques in the brains of Alzheimer disease patients’, sequential cleavage by alpha-secretase and gamma-secretase results in the formation of benign p3 fragments, which suppress amyloid-beta production [[32](#_ENREF_32)]. Microglial alpha-secretase and gamma-secretase mediate immune responses that are linked to Alzheimer disease, such as phagocytosis, release of proinflammatory cytokines and chemokines, and clearance of amyloid beta [[15](#_ENREF_15), [32-34](#_ENREF_32)].

Adcy7, Clic4, Comt1, Comtd1, Epb4.1l2, Flna, Flnb, Gnai2, Maoa, Stx3, Vamp2, Vamp3, and Vamp8 were annotated with the dopamine receptor-mediated signaling pathway. Although dopamine and dopamine receptor-mediated signaling mediate various neuronal functions [[24](#_ENREF_24)], recent studies have demonstrated that dopamine receptor in microglia facilitates chemotactic targeting and phagocytic activation that are associated with the pathogenic mechanisms of Parkinson disease [[35](#_ENREF_35)].

***Membrane proteome and N-glycoproteome in Toll-like receptor-mediated signaling pathways***

To examine the immunological function of membrane proteins and N-glycosylation that we identified, we focused on Toll-like receptor (TLR)-mediated signaling, based on the results of the bioinformatics analysis. The TLR family is a major class of pathogen-associated molecular pattern (PAMP) recognition receptors (PRRs) that trigger and tailor innate and subsequent adaptive immune responses [[36](#_ENREF_36), [37](#_ENREF_37)]. Based on sequence homology, vertebrate TLRs are grouped into 6 major families: TLR1/2/6/10, TLR3, TLR4, TLR5, TLR7/8/9, and TLR11/12/13/21/22/23 [[38](#_ENREF_38)].

Of the TLRs that were identified in our study, TLR2, in heteromeric association with TLR1 or TLR6, conveys signals to bacterial lipopeptides and peptidoglycan [[39](#_ENREF_39), [40](#_ENREF_40)]. TLR3 recognizes double-stranded RNA, which is associated with viral infection. TLR4 is activated by lipopolysaccharide (LPS), found in most gram-negative bacterial strains. TLR7 and TLR8 are sensors of virus-specific single-stranded RNA in endosomes, and TLR9 mediates cellular responses to bacterial and viral unmethylated CpG DNA [[41](#_ENREF_41)]. The ligands of novel TLRs, such as Tlr13, have not been determined.

Despite the wide range of ligands that are recognized by them, TLRs have a common structural framework, consisting of N-terminal ectodomains (ECDs), a single transmembrane helix, and a C-terminal cytoplasmic signaling domain, as shown in Supplementary Figure S9A [[42](#_ENREF_42), [43](#_ENREF_43)]. The ECDs of all TLRs typically contain 19-25 tandem copies of a motif, known as the leucine-rich repeat (LRR), which forms a horseshoe structure. In addition, TLR-ECDs are heavily glycosylated and constructed of structures that cap the N- and C-terminal ends, known as the LRR-NT and LRR-CT motifs, respectively [[43](#_ENREF_43)]. The C-terminal cytoplasmic signaling domain is known as the Toll IL-1 receptor (TIR) domain [[44](#_ENREF_44)]. Although recent structural studies of TLRs have demonstrated that the structure and mode of TLR-ligand interactions is reciprocally similar, the ECDs of vertebrate TLRs vary in the numbers of LRRs that they contain and the extent of N-linked glycosylation, indicating that the number of LRR motifs and their N-glycosylation are important factors in ligand binding. In particular, N-glycosylation could modulate the surface properties of ECDs that recognize ligands, affecting ligand binding.

To date, 13 TLRs have been identified in human and mouse, except for TLR10, which is expressed only in human [[19](#_ENREF_19), [45](#_ENREF_45), [46](#_ENREF_46)]. Of the proteins in the mouse TLR family, Tlr1, Tlr2, Tlr4, Tlr7, Tlr9, and Tlr13 were detected in our crude membrane proteome. In addition, numerous N-glycosites in Tlr1, Tlr4, Tlr7, Tlr9, and Tlr13 were identified in our N-glycoproteome. As shown in Supplementary Figure S9B, many N-glycosylation sites of TLRs have not been reported in existing databases or the literature.

To examine the function of the N-glycosylated sites in our data, we predicted the localization of N-glycosylated asparagine in TLRs using sequence alignment, the Uniprot database [[2](#_ENREF_2)], and the SMART server [[47](#_ENREF_47)]. As expected, most N-glycosylation sites in TLRs lay in the LRR motif in ECDs (Supplementary Figures S9A and B), consistent with other structural studies. For example, 14 N-glycosylation sites were newly identified in Tlr13 in our study, 12 of which resided in LRR motifs; N93 and N742 were located in the LRR-NT and LRR-CT motifs, respectively (Supplementary Figure S9A). We presume that novel N-glycosylation sites in TLRs govern ligand recognition and regulate TLR-mediated signaling events.

Next, to determine whether our crude membrane proteome and N-glycoproteome were preferentially enriched in TLR-mediated signaling components, we constructed an interaction network of TLR pathways using InnateDB [[48](#_ENREF_48)] and GO annotation for cellular localization (Supplementary Figure S9C). Typical TLRs are coupled to the MyD88-dependent pathway, involving the early phase of NF-κB activation, or MyD88-independent pathway, which regulates interferon regulatory factor IRF3 and the late phase of NF-κB activation, resulting in the production of proinflammatory cytokines and chemokines through the transregulation of various genes [[15](#_ENREF_15), [39](#_ENREF_39), [49](#_ENREF_49)]. Several proteins and N-glycosylation sites in TLR-mediated signaling were identified in our study. Notably, the interaction map showed many nuclear proteins that interacted with membrane proteins directly or indirectly.

As discussed, TLRs control microglial activation and microglia-mediated immune responses in the brain. For example, TLR2 is important for microglial activation following damage to sensory neurons [[45](#_ENREF_45)]. Several TLR3 and TLR4 knockdown and knockout experiments have indicated that TLR3- and TLR4-mediated responses mediate microglial activation [[15](#_ENREF_15), [22](#_ENREF_22)]. In murine microglia, TLR 9 responds to unmethylated CpG-DNA by effecting the secretion of proinflammatory cytokines, the upregulation of costimulatory cell surface molecules, and the promotion of adaptive activation through IL-12 secretion to direct T cell activation [[50](#_ENREF_50)].

TLRs also regulate microglial apoptosis following pathological activation in the brain. Recent studies have noted that TLR2 and TLR4 trigger microglial apoptosis through distinct apoptotic pathways [[51](#_ENREF_51)]. Thus, it is possible that TLR-regulated microglial cell death is an intrinsic mechanism that prevents the overactivation of microglia [[15](#_ENREF_15), [22](#_ENREF_22)]. In addition, numerous studies have demonstrated the significance of microglial TLRs in various CNS diseases, including infection, trauma, stroke, neurodegeneration, and autoimmunity. Particularly, TLRs in the CNS are linked to neurodegenerative diseases, such as Alzheimer disease, multiple sclerosis, Parkinson disease, and ALS [[22](#_ENREF_22)]. For example, in Alzheimer disease, the direct link between neurodegenerative processes and microglial TLRs were established. Microglia responded to fibrillar Aβ through the co-receptor CD14, which functions with TLR4 and TLR2 to bind fibrillar Aβ and induce microglial activation through p38 MAPK [[21](#_ENREF_21)]. Also, Aβ initiates the inflammatory response in microglia through the assembly of TLR4-TLR6 heterodimers, which is regulated by CD36 [[52](#_ENREF_52)]. Other TLRs, such as TLR7 and TLR9, regulate microglial responses, which mediate brain inflammation and neurodegenerative diseases [[53](#_ENREF_53)].

Thus, our identification of proteins and their N-glycosylation sites, especially in TLR signaling, allows one to construct a network of TLR signaling circuitry during immune responses and neurodegenerative processes that are mediated by microglia.

**Supplementary Table Legends**

**Supplementary Table S1. Summary of crude membrane proteome of microglia BV-2 cells.** Information on mass spectrometric analysis of 2 biological sets is provided. Information on transmembrane domain (TMD) prediction and GPI anchor prediction is provided, as are inform GO annotation data using the DAVID tool, UniprotKB database, and Cytoscape. Peptide spectrum match (PSM), unique peptide, protein group, average of absolute mass deviation, and mass standard deviation are listed by experimental scheme.

**Supplementray Table S2. Proteins identified in biological set 1.**

Complete list of protein groups identified in biological set 1 is shown. Leading proteins in the corresponding protein groups are also shown. List includes IPI accession number, Uniprot accession number, gene symbol, and protein name of each identified protein group. Search results from the Maxquant program-Andromeda engine are also shown. Information on transmembrane domain (TMD) prediction and GPI anchor prediction is provided, as are inform GO annotation data using the DAVID tool, UniprotKB database, and Cytoscape.

**Supplementary Table S3. Proteins identified in biological set 2.**

Complete list of protein groups identified in biological set 2 is shown. Leading proteins in the corresponding protein groups are also listed. List includes IPI accession number, Uniprot accession number, gene symbol, and protein name of each identified protein group. Search results from the Maxquant program-Andromeda engine are also shown. Data for transmembrane domain prediction and GPI anchor prediction are provided, as is information on GO annotation using the DAVID tool, UniprotKB database, and Cytoscape.

**Supplementary Table S4. N-glycoproteome by WCC at FDR < 1%.**

All identified N-glycosites by WCC at an FDR < 1% are listed. Search results derived from Maxquant-Andromeda are provided.

**Supplementary Table S5. N-glycosylation sites containing N!P-[S/T/rarely C] motif by WCC.**

All identified N-glycosites containing the N!P-[S/T/rarely C] motif by WCC are listed. Search results derived from Maxquant-Andromeda are provided.

**Supplementary Table S6. N-glycoproteome by CMC at FDR < 1%.**

All identified N-glycopeptides by CMC at an FDR < 1% are listed. Search results derived from Sequest-TPP are provided. In addition, sequence analysis was performed to examine the NxSTC motif.

**Supplementary Table S7. N-glycosylated peptides containing N!P-[S/T/rarely C] motif by CMC.**

All identified N-glycopeptides containing the N!P-[S/T/rarely C] motif in CMC are listed. Search results derived from Sequest-TPP are provided.

**Supplementary Table S8. Unique N-glycosylation sites.**

Unique N-glycosylation sites identified by WCC and CMC are listed. N-glycosites identified from both WCC and CMC are shown as the overlapping portion. Transmembrane domain prediction and GO annotation of identified glycoproteins are provided. In addition, results of the comparison analysis with UniprotDB and PHOSIDA are shown. Compared with UniprotDB, N-glycosylation sites identified only in our data are listed as "new." Among "new" N-glycosylation sites, we excluded N-glycosites annotated as "By similarity." ND: not detected.

**Supplementary Table S9. PANTHER molecular function analysis.**

Three groups of the proteome—crude membrane proteome, TMD-containing proteome, and N-glycoproteome—were subjected to PANTHER molecular function analysis. Information on molecular function terms and enrichment statistics is provided.

**Supplementary Table S10. KEGG pathway analysis.**

Three groups of the proteome—crude membrane proteome, TMD-containing proteome, and N-glycoproteome—were subjected to KEGG pathway analysis. Information on pathway terms and enrichment statistics is provided.

**Supplementary Table S11. PANTHER pathway analysis.**

Three groups of the proteome—crude membrane proteome, TMD-containing proteome, and N-glycoproteome—were subjected to PANTHER pathway analysis. Information on pathway terms and enrichment statistics is provided.

**Supplementary References**

1. Huang da W, Sherman BT, Lempicki RA: **Systematic and integrative analysis of large gene lists using DAVID bioinformatics resources**. *Nature protocols* 2009, **4**(1):44-57.

2. Magrane M, Consortium U: **UniProt Knowledgebase: a hub of integrated protein data**. *Database : the journal of biological databases and curation* 2011, **2011**:bar009.

3. Dimmer EC, Huntley RP, Alam-Faruque Y, Sawford T, O'Donovan C, Martin MJ, Bely B, Browne P, Mun Chan W, Eberhardt R *et al*: **The UniProt-GO Annotation database in 2011**. *Nucleic acids research* 2012, **40**(Database issue):D565-570.

4. Shannon P, Markiel A, Ozier O, Baliga NS, Wang JT, Ramage D, Amin N, Schwikowski B, Ideker T: **Cytoscape: a software environment for integrated models of biomolecular interaction networks**. *Genome research* 2003, **13**(11):2498-2504.

5. Maere S, Heymans K, Kuiper M: **BiNGO: a Cytoscape plugin to assess overrepresentation of gene ontology categories in biological networks**. *Bioinformatics* 2005, **21**(16):3448-3449.

6. Thomas PD, Kejariwal A, Campbell MJ, Mi H, Diemer K, Guo N, Ladunga I, Ulitsky-Lazareva B, Muruganujan A, Rabkin S *et al*: **PANTHER: a browsable database of gene products organized by biological function, using curated protein family and subfamily classification**. *Nucleic acids research* 2003, **31**(1):334-341.

7. Hosack DA, Dennis G, Jr., Sherman BT, Lane HC, Lempicki RA: **Identifying biological themes within lists of genes with EASE**. *Genome Biol* 2003, **4**(10):R70.

8. Fankhauser N, Maser P: **Identification of GPI anchor attachment signals by a Kohonen self-organizing map**. *Bioinformatics* 2005, **21**(9):1846-1852.

9. Pierleoni A, Martelli PL, Casadio R: **PredGPI: a GPI-anchor predictor**. *BMC bioinformatics* 2008, **9**:392.

10. Emanuelsson O, Nielsen H, Brunak S, von Heijne G: **Predicting subcellular localization of proteins based on their N-terminal amino acid sequence**. *Journal of molecular biology* 2000, **300**(4):1005-1016.

11. Cox J, Mann M: **MaxQuant enables high peptide identification rates, individualized p.p.b.-range mass accuracies and proteome-wide protein quantification**. *Nature biotechnology* 2008, **26**(12):1367-1372.

12. Keller A, Nesvizhskii AI, Kolker E, Aebersold R: **Empirical statistical model to estimate the accuracy of peptide identifications made by MS/MS and database search**. *Analytical chemistry* 2002, **74**(20):5383-5392.

13. Lu B, McClatchy DB, Kim JY, Yates JR, 3rd: **Strategies for shotgun identification of integral membrane proteins by tandem mass spectrometry**. *Proteomics* 2008, **8**(19):3947-3955.

14. Vuckovic D, Dagley LF, Purcell AW, Emili A: **Membrane proteomics by high performance liquid chromatography - tandem mass spectrometry: analytical approaches and challenges**. *Proteomics* 2012.

15. Kettenmann H, Hanisch UK, Noda M, Verkhratsky A: **Physiology of microglia**. *Physiological reviews* 2011, **91**(2):461-553.

16. Goddard DR, Berry M, Kirvell SL, Butt AM: **Fibroblast growth factor-2 induces astroglial and microglial reactivity in vivo**. *Journal of anatomy* 2002, **200**(Pt 1):57-67.

17. Qu WS, Tian DS, Guo ZB, Fang J, Zhang Q, Yu ZY, Xie MJ, Zhang HQ, Lu JG, Wang W: **Inhibition of EGFR/MAPK signaling reduces microglial inflammatory response and the associated secondary damage in rats after spinal cord injury**. *Journal of neuroinflammation* 2012, **9**:178.

18. Colton CA: **Heterogeneity of microglial activation in the innate immune response in the brain**. *Journal of neuroimmune pharmacology : the official journal of the Society on NeuroImmune Pharmacology* 2009, **4**(4):399-418.

19. Olson JK, Miller SD: **Microglia initiate central nervous system innate and adaptive immune responses through multiple TLRs**. *Journal of immunology* 2004, **173**(6):3916-3924.

20. Cerri C, Fabbri A, Vannini E, Spolidoro M, Costa M, Maffei L, Fiorentini C, Caleo M: **Activation of Rho GTPases triggers structural remodeling and functional plasticity in the adult rat visual cortex**. *The Journal of neuroscience : the official journal of the Society for Neuroscience* 2011, **31**(42):15163-15172.

21. Reed-Geaghan EG, Savage JC, Hise AG, Landreth GE: **CD14 and toll-like receptors 2 and 4 are required for fibrillar A{beta}-stimulated microglial activation**. *The Journal of neuroscience : the official journal of the Society for Neuroscience* 2009, **29**(38):11982-11992.

22. Okun E, Griffioen KJ, Lathia JD, Tang SC, Mattson MP, Arumugam TV: **Toll-like receptors in neurodegeneration**. *Brain research reviews* 2009, **59**(2):278-292.

23. Qian L, Wu HM, Chen SH, Zhang D, Ali SF, Peterson L, Wilson B, Lu RB, Hong JS, Flood PM: **beta2-adrenergic receptor activation prevents rodent dopaminergic neurotoxicity by inhibiting microglia via a novel signaling pathway**. *Journal of immunology* 2011, **186**(7):4443-4454.

24. Beaulieu JM, Gainetdinov RR: **The physiology, signaling, and pharmacology of dopamine receptors**. *Pharmacological reviews* 2011, **63**(1):182-217.

25. Moller T, Kann O, Prinz M, Kirchhoff F, Verkhratsky A, Kettenmann H: **Endothelin-induced calcium signaling in cultured mouse microglial cells is mediated through ETB receptors**. *Neuroreport* 1997, **8**(9-10):2127-2131.

26. Grandbarbe L, Michelucci A, Heurtaux T, Hemmer K, Morga E, Heuschling P: **Notch signaling modulates the activation of microglial cells**. *Glia* 2007, **55**(15):1519-1530.

27. Wei Z, Chigurupati S, Arumugam TV, Jo DG, Li H, Chan SL: **Notch activation enhances the microglia-mediated inflammatory response associated with focal cerebral ischemia**. *Stroke; a journal of cerebral circulation* 2011, **42**(9):2589-2594.

28. Ables JL, Breunig JJ, Eisch AJ, Rakic P: **Not(ch) just development: Notch signalling in the adult brain**. *Nature reviews Neuroscience* 2011, **12**(5):269-283.

29. Tanzi RE, Bertram L: **Twenty years of the Alzheimer's disease amyloid hypothesis: a genetic perspective**. *Cell* 2005, **120**(4):545-555.

30. Takasugi N, Tomita T, Hayashi I, Tsuruoka M, Niimura M, Takahashi Y, Thinakaran G, Iwatsubo T: **The role of presenilin cofactors in the gamma-secretase complex**. *Nature* 2003, **422**(6930):438-441.

31. Shah S, Lee SF, Tabuchi K, Hao YH, Yu C, LaPlant Q, Ball H, Dann CE, 3rd, Sudhof T, Yu G: **Nicastrin functions as a gamma-secretase-substrate receptor**. *Cell* 2005, **122**(3):435-447.

32. De Strooper B, Vassar R, Golde T: **The secretases: enzymes with therapeutic potential in Alzheimer disease**. *Nature reviews Neurology* 2010, **6**(2):99-107.

33. Farfara D, Trudler D, Segev-Amzaleg N, Galron R, Stein R, Frenkel D: **gamma-Secretase component presenilin is important for microglia beta-amyloid clearance**. *Annals of neurology* 2011, **69**(1):170-180.

34. Pan XD, Zhu YG, Lin N, Zhang J, Ye QY, Huang HP, Chen XC: **Microglial phagocytosis induced by fibrillar beta-amyloid is attenuated by oligomeric beta-amyloid: implications for Alzheimer's disease**. *Molecular neurodegeneration* 2011, **6**:45.

35. Mastroeni D, Grover A, Leonard B, Joyce JN, Coleman PD, Kozik B, Bellinger DL, Rogers J: **Microglial responses to dopamine in a cell culture model of Parkinson's disease**. *Neurobiology of aging* 2009, **30**(11):1805-1817.

36. Beutler B: **Inferences, questions and possibilities in Toll-like receptor signalling**. *Nature* 2004, **430**(6996):257-263.

37. Iwasaki A, Medzhitov R: **Toll-like receptor control of the adaptive immune responses**. *Nature immunology* 2004, **5**(10):987-995.

38. Roach JC, Glusman G, Rowen L, Kaur A, Purcell MK, Smith KD, Hood LE, Aderem A: **The evolution of vertebrate Toll-like receptors**. *Proceedings of the National Academy of Sciences of the United States of America* 2005, **102**(27):9577-9582.

39. Akira S, Takeda K: **Toll-like receptor signalling**. *Nature reviews Immunology* 2004, **4**(7):499-511.

40. Jin MS, Kim SE, Heo JY, Lee ME, Kim HM, Paik SG, Lee H, Lee JO: **Crystal structure of the TLR1-TLR2 heterodimer induced by binding of a tri-acylated lipopeptide**. *Cell* 2007, **130**(6):1071-1082.

41. Hemmi H, Takeuchi O, Kawai T, Kaisho T, Sato S, Sanjo H, Matsumoto M, Hoshino K, Wagner H, Takeda K *et al*: **A Toll-like receptor recognizes bacterial DNA**. *Nature* 2000, **408**(6813):740-745.

42. Bell JK, Mullen GE, Leifer CA, Mazzoni A, Davies DR, Segal DM: **Leucine-rich repeats and pathogen recognition in Toll-like receptors**. *Trends in immunology* 2003, **24**(10):528-533.

43. Botos I, Segal DM, Davies DR: **The structural biology of Toll-like receptors**. *Structure* 2011, **19**(4):447-459.

44. O'Neill LA, Bowie AG: **The family of five: TIR-domain-containing adaptors in Toll-like receptor signalling**. *Nature reviews Immunology* 2007, **7**(5):353-364.

45. Jack CS, Arbour N, Manusow J, Montgrain V, Blain M, McCrea E, Shapiro A, Antel JP: **TLR signaling tailors innate immune responses in human microglia and astrocytes**. *Journal of immunology* 2005, **175**(7):4320-4330.

46. Mishra BB, Gundra UM, Teale JM: **Expression and distribution of Toll-like receptors 11-13 in the brain during murine neurocysticercosis**. *Journal of neuroinflammation* 2008, **5**:53.

47. Letunic I, Copley RR, Schmidt S, Ciccarelli FD, Doerks T, Schultz J, Ponting CP, Bork P: **SMART 4.0: towards genomic data integration**. *Nucleic acids research* 2004, **32**(Database issue):D142-144.

48. Lynn DJ, Winsor GL, Chan C, Richard N, Laird MR, Barsky A, Gardy JL, Roche FM, Chan TH, Shah N *et al*: **InnateDB: facilitating systems-level analyses of the mammalian innate immune response**. *Molecular systems biology* 2008, **4**:218.

49. Jin MS, Lee JO: **Structures of the toll-like receptor family and its ligand complexes**. *Immunity* 2008, **29**(2):182-191.

50. Dalpke AH, Schafer MK, Frey M, Zimmermann S, Tebbe J, Weihe E, Heeg K: **Immunostimulatory CpG-DNA activates murine microglia**. *Journal of immunology* 2002, **168**(10):4854-4863.

51. Tang SC, Arumugam TV, Xu X, Cheng A, Mughal MR, Jo DG, Lathia JD, Siler DA, Chigurupati S, Ouyang X *et al*: **Pivotal role for neuronal Toll-like receptors in ischemic brain injury and functional deficits**. *Proceedings of the National Academy of Sciences of the United States of America* 2007, **104**(34):13798-13803.

52. Stewart CR, Stuart LM, Wilkinson K, van Gils JM, Deng J, Halle A, Rayner KJ, Boyer L, Zhong R, Frazier WA *et al*: **CD36 ligands promote sterile inflammation through assembly of a Toll-like receptor 4 and 6 heterodimer**. *Nature immunology* 2010, **11**(2):155-161.

53. Tahara K, Kim HD, Jin JJ, Maxwell JA, Li L, Fukuchi K: **Role of toll-like receptor signalling in Abeta uptake and clearance**. *Brain : a journal of neurology* 2006, **129**(Pt 11):3006-3019.
