# Supplementary material for: Characterization of the membrane proteome and N-glycoproteome in BV-2 mouse microglia by liquid chromatography-tandem mass spectrometry
Source: BMC Genomics. 2014 Feb 4;15:95. doi: 10.1186/1471-2164-15-95 (PMC3938046; doi:10.1186/1471-2164-15-95)
Supplement: Additional file 4: Figure S1 — Detailed flowchart for the identification of membrane proteins and N-glycoproteins. Figure S2. Technical and biological reproducibility in experiments for crude membrane proteome. Figure S3. Complementarity of multiple strategies for comprehensive coverage of crude membrane proteome. Figure S4. Comparison between biological replicates by WCC and CMC for the N-glycoproteome. Figure S5. Technical reproducibility and biological reproducibility in experiments for N-glycoproteome profiling. Figure S6. Comparison of BV-2 crude membrane proteome and N-glycoproteome. Figure S7. Comparison with transcriptomics data to identify microglia-specific glycoproteins. Figure S8. Characterization of TMD-containing proteins and N-glycoproteins. Figure S9. Detailed information on Toll-like receptor (TLR) family. [file 1471-2164-15-95-S4.docx]

**Supplementary Figures**

**Figure S1**

**
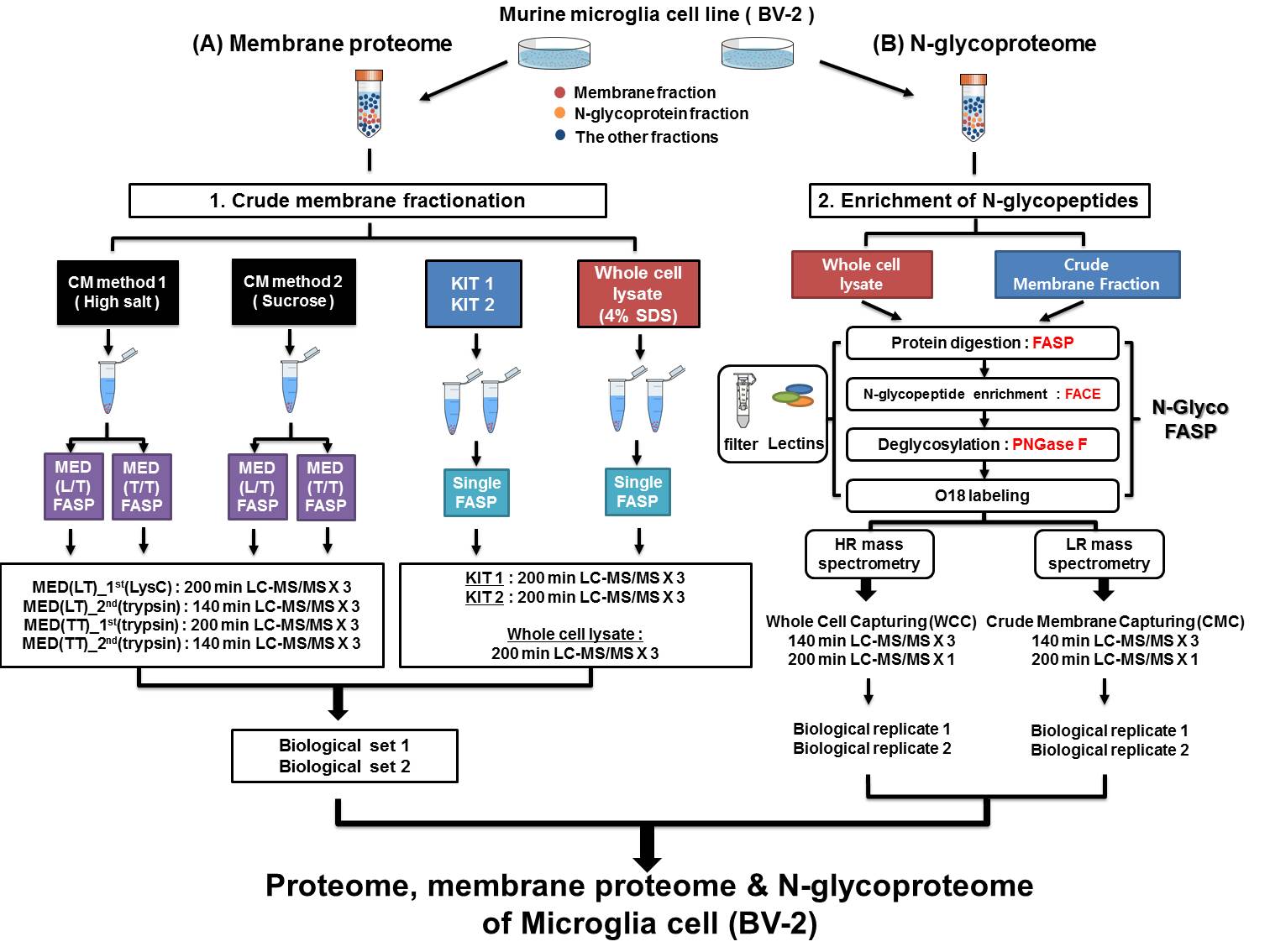
**

**Supplementary Figure S1. Detailed flowchart for the identification of membrane proteins and N-glycoproteins**

Crude membrane fractionation and N-glyco-FASP were performed. BV-2 cells were lysed and fractionated using CM method 1, CM method 2, KIT 1, KIT 2, and 4% SDS. Fractionated proteins were digested by FASP or MED-FASP, separated on a reverse-phase column, and analyzed by LC-MS/MS, using MS2-TOP10 methodology by high-resolution (HR) mass spectrometry (Orbitrap Velos and Q Exactive). For the N-glycoproteome of BV-2 cells, whole-cell lysate and crude membrane fractions were subjected to N-glyco-FASP. Glycopeptides obtained from whole cell capturing (WCC) were analyzed by high-resolution mass spectrometry (Orbitrap Velos and Q Exactive), whereas glycopeptides obtained from crude membrane fraction capturing (CMC) were analyzed by low-resolution mass spectrometry (LTQ Velos). Detailed schemes for the LC-MS/MS analysis are shown, including the number of technical replicates and biological replicates and LC gradient time.

**Figure S2**

**
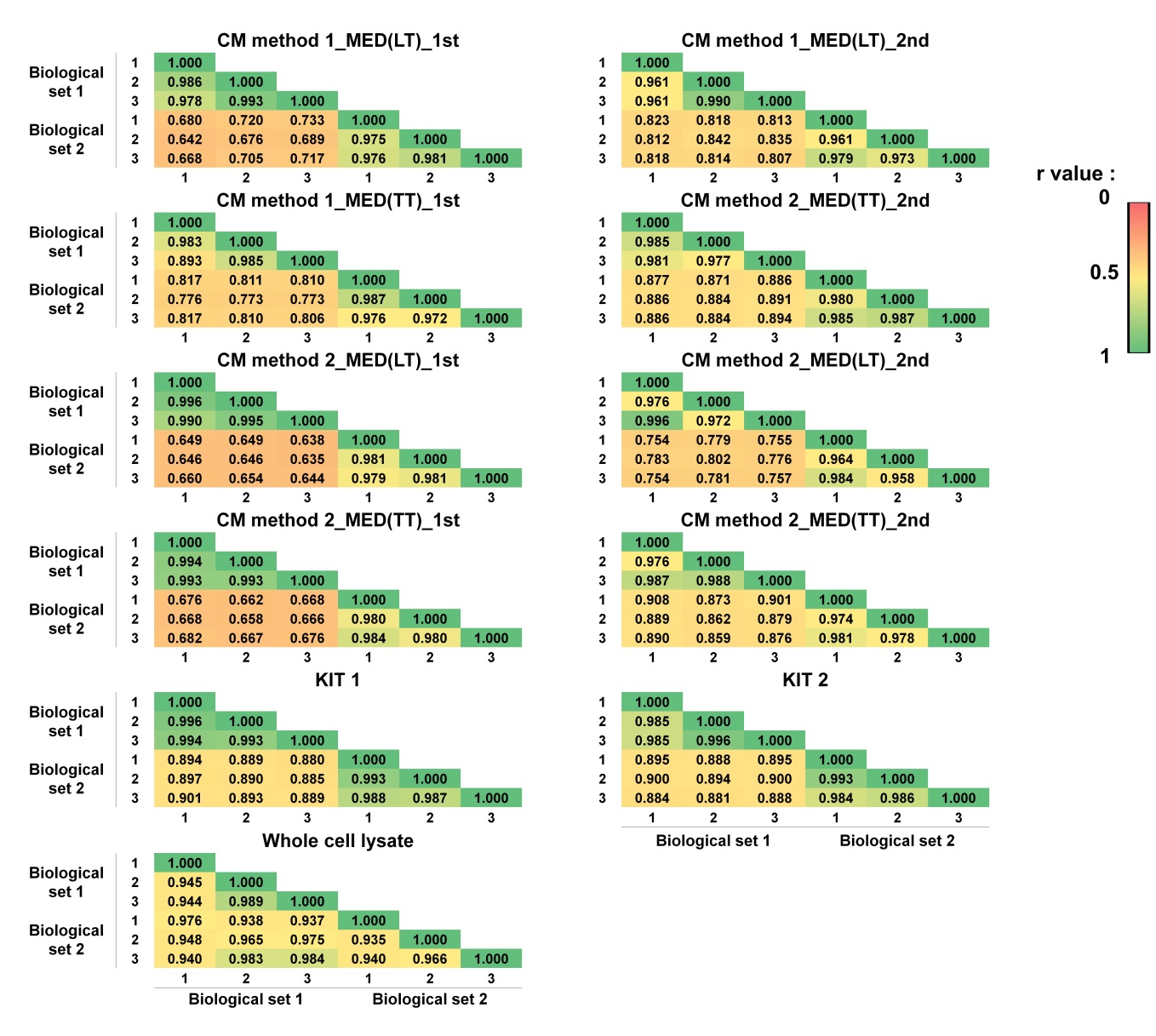
**

**Supplementary Figure S2. Technical and biological reproducibility in experiments for crude membrane proteome**

Pearson correlation coefficients (r values) were calculated to determine the reproducibility across technical and biological replicates. Two combinations (LysC/trypsin and trypsin/trypsin) were termed LT and TT, respectively. In each experiment, the first and second digestion datasets were displayed as 1st and 2^nd^ for the 2 combinations.

**Figure S3**

**
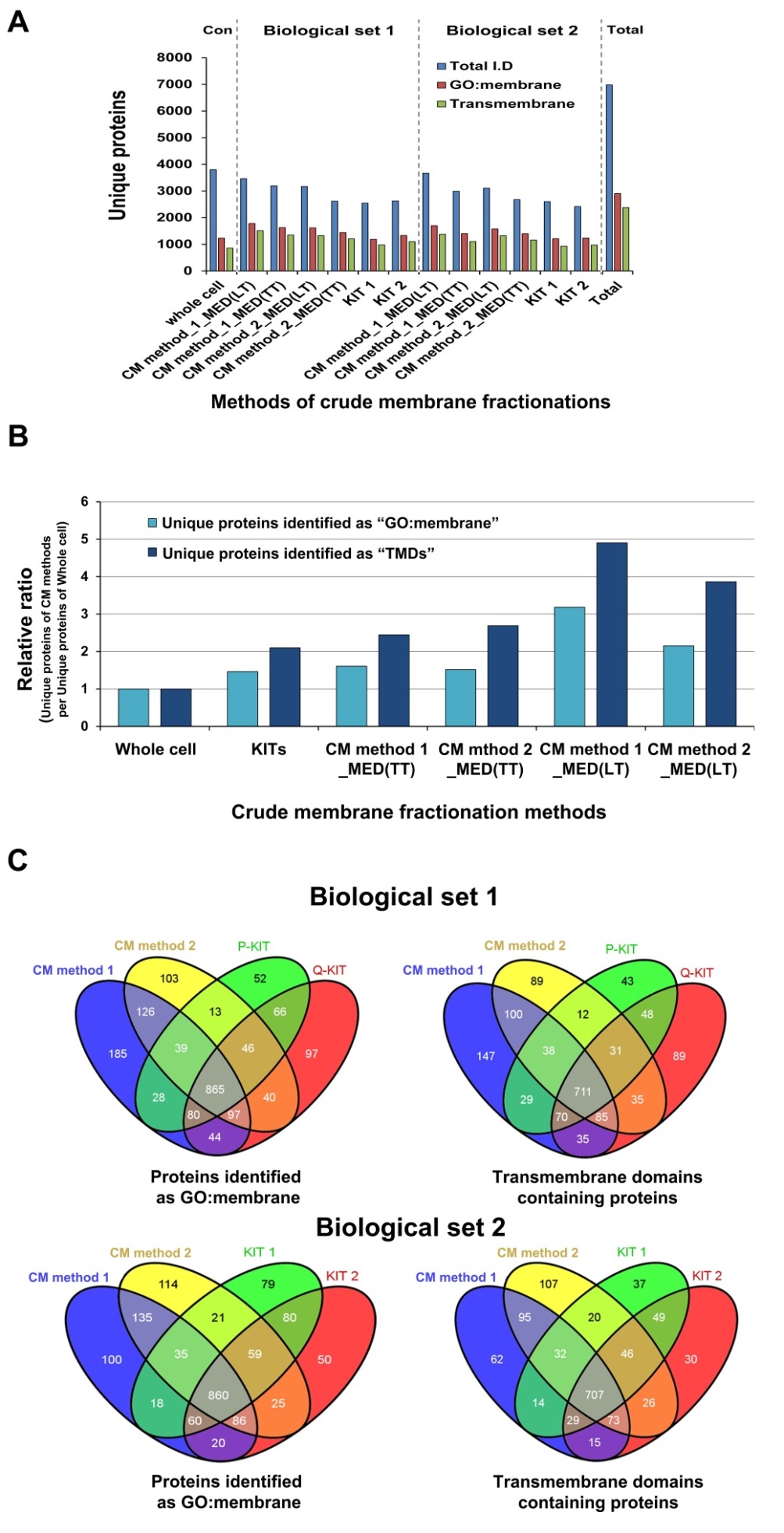
**

**Supplementary Figure S3. Complementarity of multiple strategies for comprehensive coverage of crude membrane proteome**

(A) Identification of total proteins, membrane proteins annotated as GO term “membrane,” and transmembrane domain-containing proteins from each method. Blue, red, and green bars indicate the numbers of total identified proteins, membrane proteins annotated as GO term “membrane,” and transmembrane domain-containing proteins, respectively. (B) The relative ratios between crude membrane fractionation methods and whole cells were compared as bar diagrams. Light blue bars indicate proteins identified as GO term “membrane,” and deep blue bars represents proteins containing transmembrane domains. (B) Coverage complementarity of crude membrane fractionation methods in biological sets 1 and 2. Proteins identified as GO term “membrane” from 4 methods are overlapped. Also, the transmembrane domains (TMD)-containing proteins obtained from the 4 methods are overlapped.

**Figure S4**


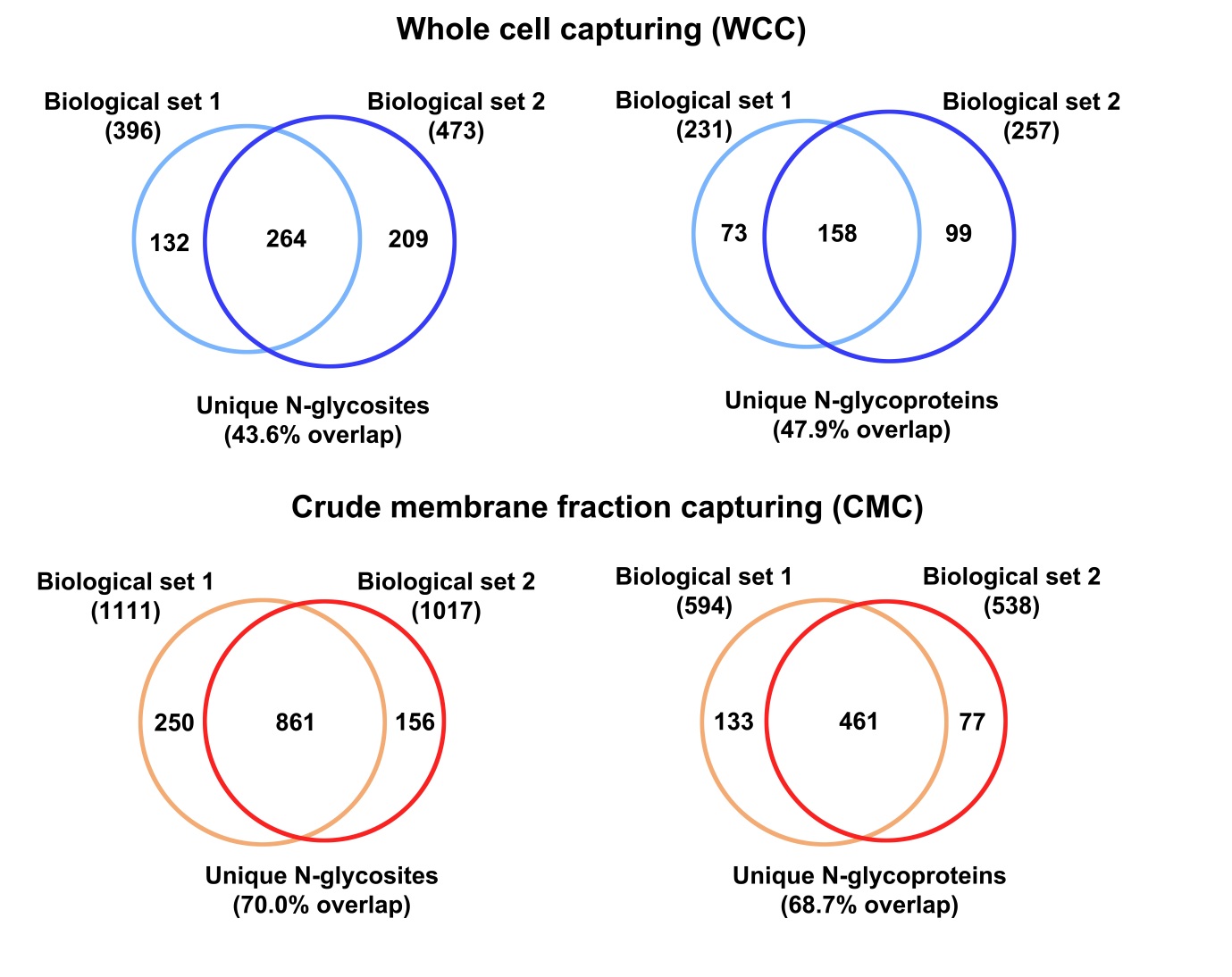


**Supplementary Figure S4. Comparison between biological replicates by WCC and CMC for the N-glycoproteome**

(A) Area-proportional Venn diagrams of the overlap in uniquely identified N-glycosites (left) and N-glycoproteins (right) between biological replicates by WCC. (B) Area-proportional Venn diagrams of the overlap in uniquely identified N-glycosites (left) and N-glycoproteins (right) between peptide preparation methods.

**Figure S5**


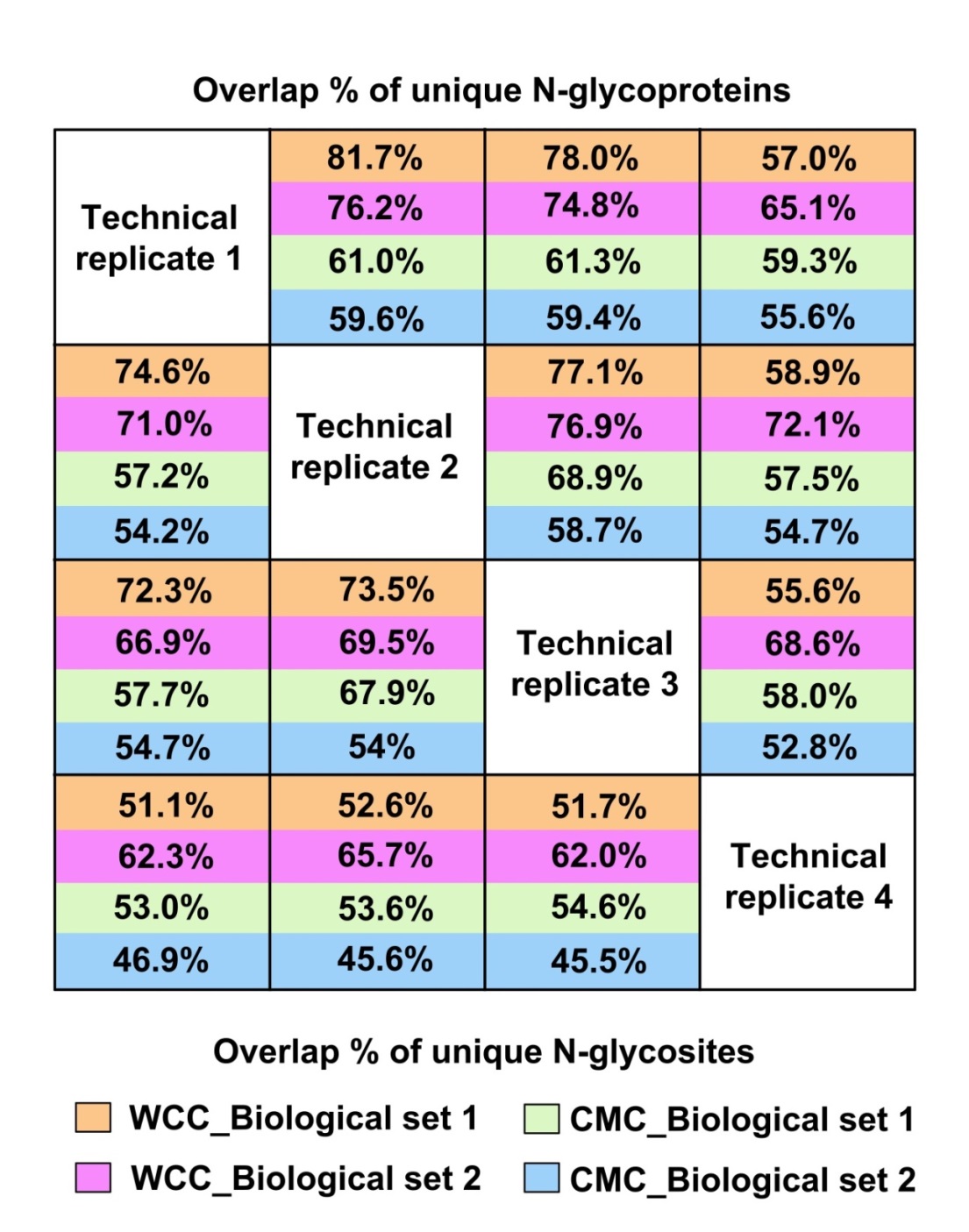


**Supplementary Figure S5. Technical reproducibility and biological reproducibility in experiments for N-glycoproteome profiling**

Overlap between datasets is represented such that the reproducibility is shown across technical and biological replicates.

**Figure S6**


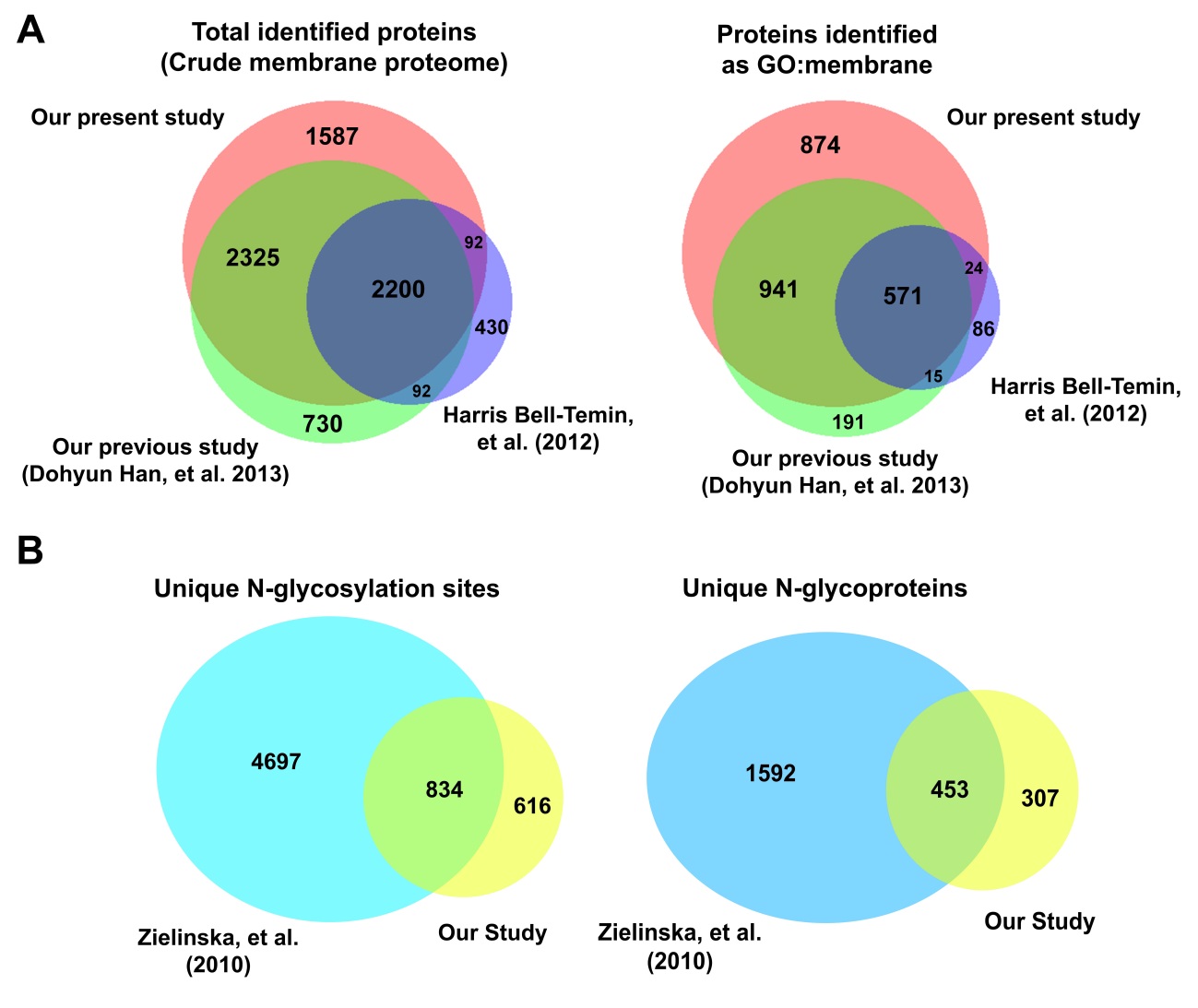


**Figure S6. Comparison of BV-2 crude membrane proteome and N-glycoproteome**

(A) Comparison between our microglia crude membrane proteome and previous studies. Accession numbers of proteins identified in this study were changed to gene symbols. Total identified proteins, except for N-glycoproteins, were compared with the previous microglia proteome (Harris Bell-Temin, et al and Dohyun Han, et al). Proteins identified as GO term “membrane” were also compared. (B) Area-proportional Venn diagrams of the overlap in uniquely identified N-glycosylation sites (A) and N-glycoproteins (B) between our proteome and the mouse N-glycoproteome of Zielinska, et al. IPI accession numbers from our results were used for this comparison.

**Supplementary Figure S7**


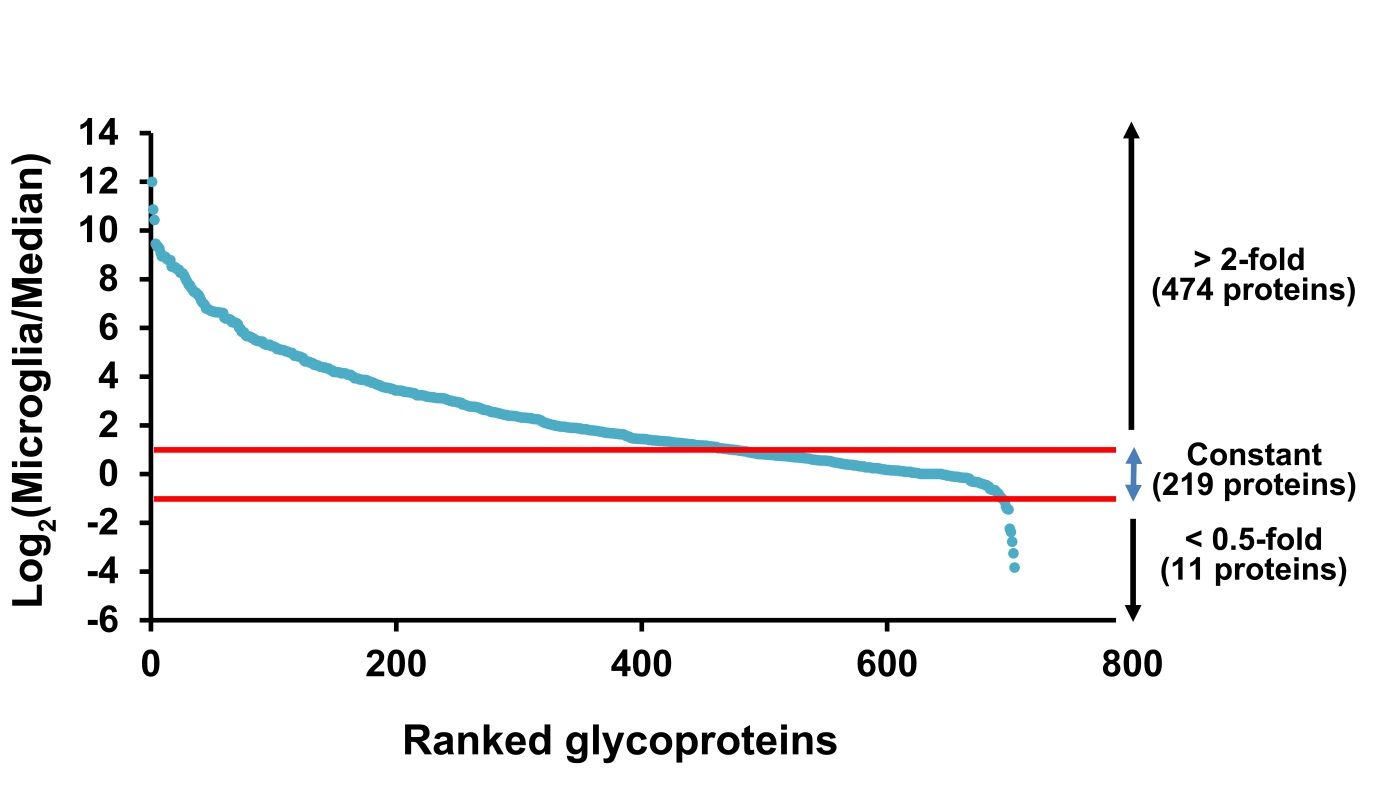


**Figure S7. Comparison with transcriptomics data to identify microglia-specific glycoproteins**

The BV-2 N-glycoproteome was compared with published mouse tissue transcriptomic data from the BioGPS server (refer to main text). Ranked N-glycoproteins are listed on the X-axis, and the Y-axis represents a scale of gene expression levels in microglia compared with 96 other mouse tissues. The Y-values were calculated as log_2_(fold change) ratio of expression in microglia versus the median value for the 96 mouse tissues. Red line demarcates a 2-fold increase and decrease in expression level in normal microglia versus all other tissues.

**Figure S8**


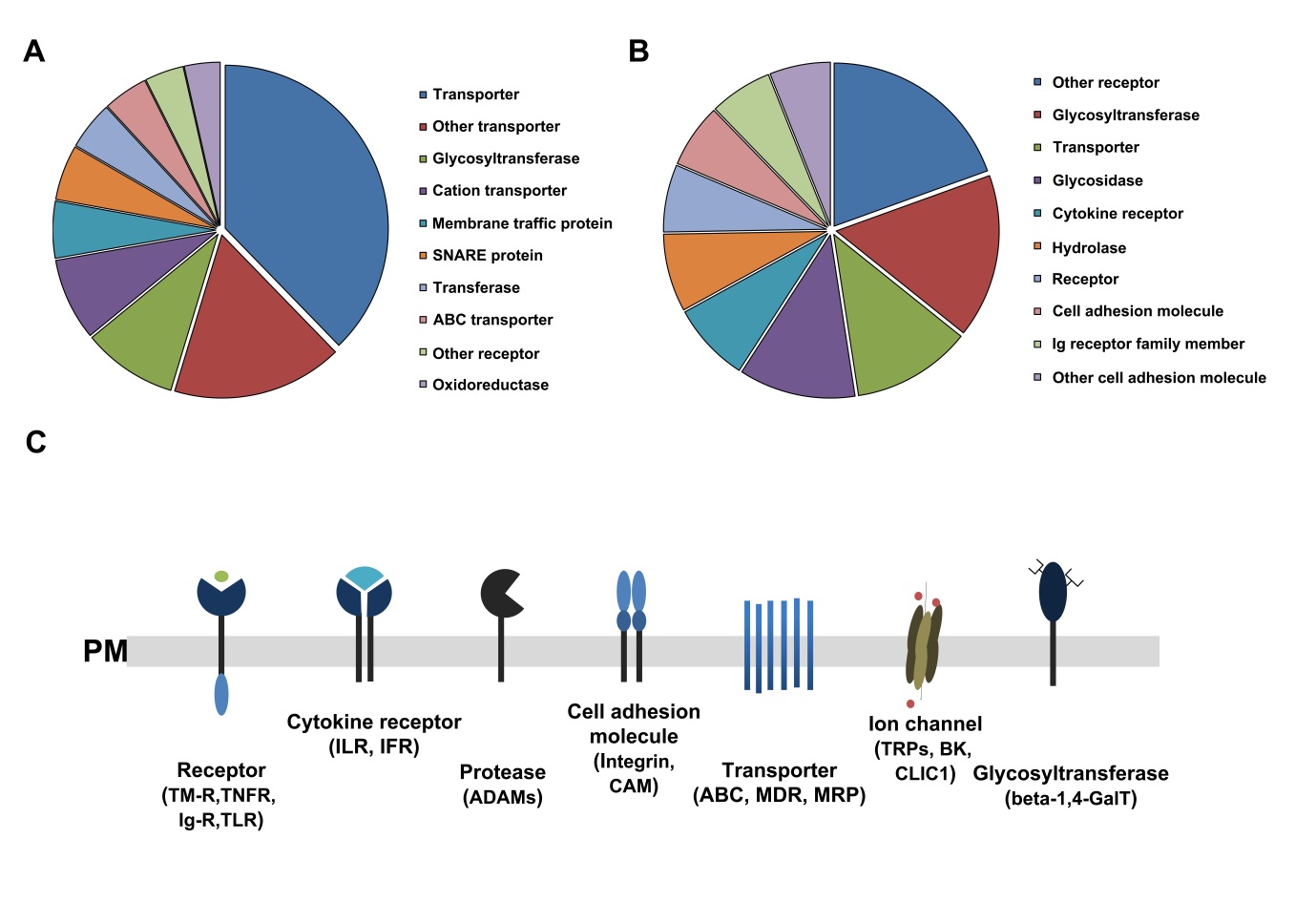


**Supplementary Figure S8. Characterization of TMD-containing proteins and N-glycoproteins**

(A) Functional categorization of TMD-containing proteins and (B) N-glycoproteins by PANTHER database based on significant overrepresentation (*p*-values) of proteins in each functional group. (C) Visualization of microglia BV-2 surface proteins based on the crude membrane proteome and N-glycoproteome. Major protein groups are shown based on their functional relationship with microglial function in the brain. Prominent proteins and protein families in each group are indicated in parentheses.

**Figure S9**


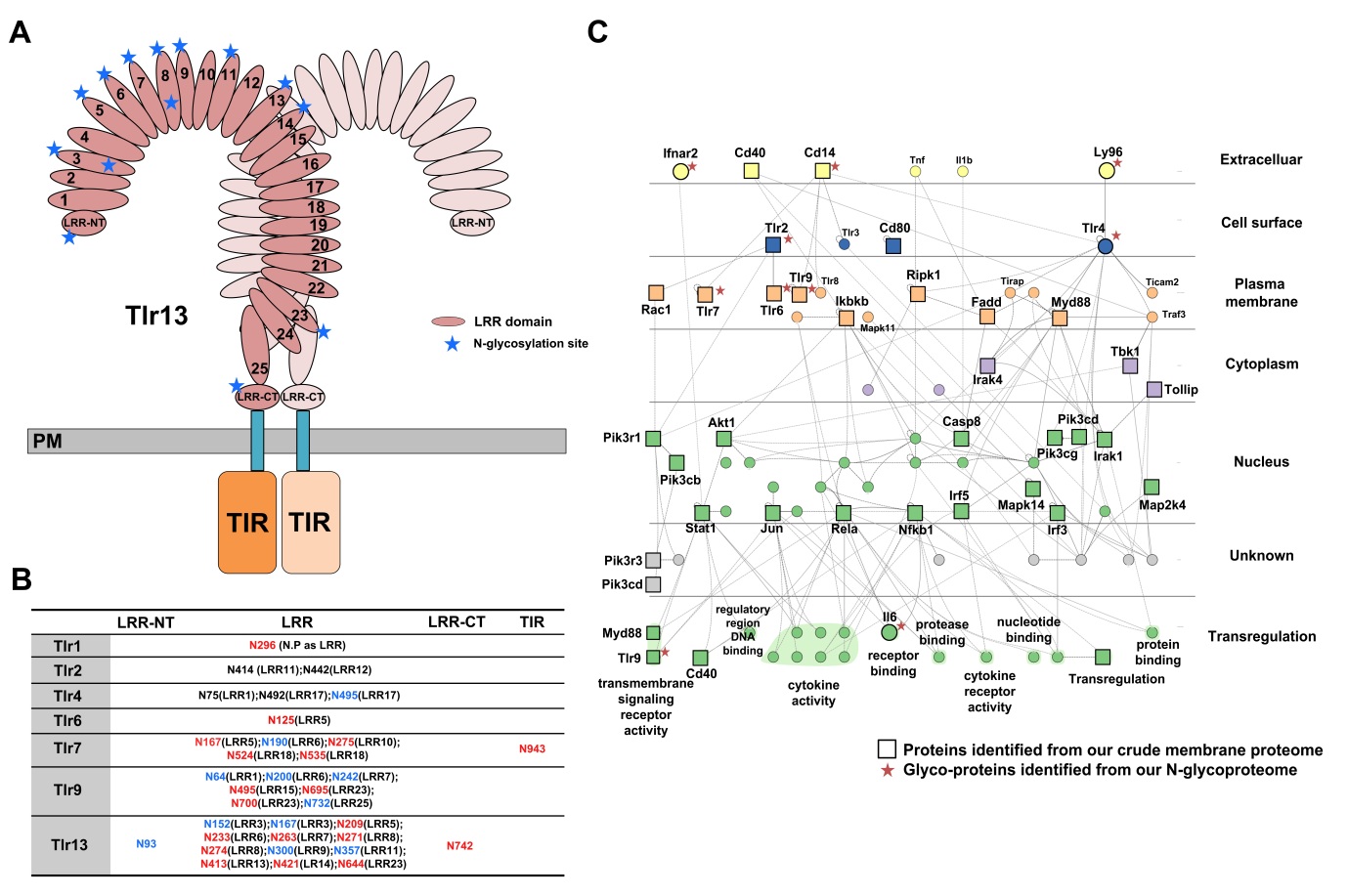


**Figure S9. Detailed information on Toll-like receptor (TLR) family**

(A) Diagram of the putative Toll-like receptor 13 dimer. The putative model of Tlr13 was constructed based on domain prediction and the crystal structure of other TLRs (refer to supplementary text). The N-glycosylation sites in our study are shown as blue asterisks. (B) Table of N-glycosylation sites in our N-glycoproteome data. The localization of N-glycosylation sites in the domains of each TLR is indicated. TLRs comprise leucine-rich repeat (LRR), N-terminal end of the LRR (LRR-NT), C-terminal end of the LRR (LRR-CT), and Toll IL-1 receptor (TIR) domains. Compared with the UniprotKB database, the novel glycosylation sites are indicated in red, and known glycosylation sites are in black. Glycosylation sites in blue are newly identified versus the PHOSIDA database (refer to main text). (C) Interaction networks of TLR signaling pathways. All proteins and their interactions with TLR signaling in mouse were extracted from the InnateDB, and a protein-protein interaction network was constructed using Cytoscape with the Cerebral plugin based on the subcellular localization of proteins. Basically, components of the interaction network are shown as circles, proteins identified from our crude membrane proteome are highlighted as squares, and N-glycoproteins observed in our N-glycoproteome are indicated as red asterisks.
